# Impact of structurally diverse polysaccharides on colonic mucin *O*-glycosylation and gut microbiota

**DOI:** 10.1038/s41522-023-00468-3

**Published:** 2023-12-11

**Authors:** Tong Zhao, Yue Zhang, Linhua Nan, Qing Zhu, Shukai Wang, Yutao Xie, Xinling Dong, Cui Cao, Xiaoliang Lin, Yu Lu, Yuxia Liu, Linjuan Huang, Guiping Gong, Zhongfu Wang

**Affiliations:** 1 Shaanxi Natural Carbohydrate Resource Engineering Research Center, College of Food Science and Technology, Northwest University, Xi’an, 710069 China; 2 Infinitus (China) Company Ltd, Guangzhou, 510000 Guangdong China

**Keywords:** Health care, Microbiome, Biofilms

## Abstract

Understanding how dietary polysaccharides affect mucin *O*-glycosylation and gut microbiota could provide various nutrition-based treatments. Here, the *O*-glycan profile of the colonic mucosa and gut microbiome were investigated in C57BL/6J mice fed six structurally diverse dietary polysaccharides and a mixture of six fibers. Dietary polysaccharides increased total *O*-glycans, mainly by stimulating neutral glycans. Highly branched arabinogalactan promoted terminally fucosylated core 1 *O*-glycans; whereas linear polysaccharides, including pectin, konjac glucomannan, inulin, and the fiber mixture, favored terminally di-fucosylated *O*-glycans. The last three polysaccharides also lowered the level of sulfated *O*-glycans and sialylated mono-fucosylated *O*-glycans. Varied monosaccharide composition in mixed polysaccharides had a synergistic beneficial effect, boosting fucosylated neutral glycans, decreasing acidic glycans, and stimulating microbial richness and diversity. Dietary polysaccharides containing arabinose and sulfate groups enhanced the relative abundances of *Akkermansia* and *Muribaculaceae*, respectively. The present comparison reveals the relationship between dietary polysaccharide structure, mucin *O*-glycan composition, and intestinal microorganisms.

## Introduction

The intestinal tract fulfills a double role by enabling the digestion and absorption of nutrients, as well as by influencing the host immune system^1–3^. Any damage to the intestinal barrier is associated with the occurrence and progression of intestinal or parenteral diseases^4^. The mucus layer lining the gut protects against potential biological, physical, and chemical attacks, while also providing adhesion sites and nutrient/energy sources in the form of specific *O*-glycans for symbiotic bacteria^1,2,4–7^.

Mucin (Muc2) is the major structural and functional constituent of the colonic mucus layer. More than 80% of its mass is constituted by *O*-glycans^8,9^. Mucin *O*-glycosylation begins with the binding of N-acetylgalactosamine (GalNAc) to the OH group of serine or threonine. The resulting Tn antigen is then extended through the addition of N-acetylglucosamine (GlcNAc), and GalNAc^10^. Core 1 Galβ1,3GalNAcα1-Ser/Thr and core 2 Galβ1,3(GlcNAcβ1,6)GalNAcα1-Ser/Thr structures are the main components of mouse Muc2^11^. The core structure of *O*-glycans is further extended through the addition of fucose (Fuc), N-acetylneuraminic acid (Neu5Ac), GalNAc, Gal, and sulfated group, forming an even more complex structure^12^.

The biological functions of mucin are closely related to its diverse *O*-glycan structure. *O*-glycans confer viscoelasticity and hydration capacity^8^. The latter imparts gel-like properties and prevents proteases from degrading the protein backbone^1^. At the same time, mucin *O*-glycans provide colonization sites for probiotics, which prevent pathogen infection through site competition^13^. Colonization of the mucus layer by *Akkermansia* stimulates the production of intestinal Muc2 and host defenses, thereby preventing the settling of other pathogenic bacteria^14,15^. Finally, mucin *O*-glycans could serve as nutrients for specific intestinal bacteria, such as *Akkermansia muciniphila, Bifidobacterium bifidum, Bacteroides thetaiotaomicron*, and *Ruminococcus torques*, with the resulting oligosaccharides or monosaccharides then cross-fed to other intestinal bacteria to maintain microbial homeostasis^16^.

Mucin *O*-glycans impact the gut microbiota, meanwhile, the gut microbes and microbial metabolite production change intestinal mucin *O*-glycans^1^. *Faecalibacterium prausnitzii* and *B. thetaiotaomicron*, regulate the levels of glycosylation-related genes (e.g., *c1galt1* and *b4galt4*) in mucin-secreting goblet cells^17^. Da Silva et al. found that oral administration of the probiotic *Lactobacillus farciminis* significantly reduced the expression of intestinal sialylated *O*-glycans (*m/z* 1331 and 1605) in irritable bowel syndrome^18^. The expression of glycosyltransferases is impacted by changes in bacterial compositions, which in turn affects the degree of mucin *O*-glycosylation. When feces from healthy mice were transplanted into germ-free mice, *Clostridia*, *Lactobacillus*, *Bacteroides*, and *Rikenellaceae* were the dominant bacteria in the mouse colon 8 weeks after the transplant, coinciding with the downregulation of St6GalNac1 and upregulation of Fut2 and C1GalT1 *O*-glycosyltransferases. This resulted in a significant decrease in sulfated *O*-glycans (*m/z* 667), but an increase in fucosylated *O*-glycans (*m/z* 975) in colonic mucin^19^. In addition, the expression of intestinal mucin *O*-glycans is influenced by short-chain fatty acids (SCFAs) generated by symbiotic microorganisms. Acetate alters mucin *O*-glycosylation by adjusting the expression of β1,3-galactosyltransferase and sialyltransferase^17^. Butyrate binds to antigen-presenting cells and induces the division of T regulatory cells, which causes the release of interleukin-10^20^. The latter then stimulates Muc2-related genes and alters the pattern of intestinal mucin *O*-glycans^21^. Therefore, this relationship is bidirectional between the gut bacteria and *O*-glycan of mucin.

Dietary intake plays a critical role in determining the composition of gut bacteria^22^. Dietary polysaccharides, which cannot be digested or absorbed in the stomach and small intestine, are fermented and metabolized by the microbiota of the colon. However, dietary polysaccharides originate from a broad range of sources and present complex chemical structures, their influence on the composition of intestinal bacteria varies^23^. Pectin from apples (RG-I) was found to favor *Faecalibaculum* and *Lactobacillus* spp. in the murine gut^24,25^; supplemented with Arabinogalactan from *Lycium barbarum* polysaccharides (LBP-3) improved the related abundance of the *Prevotellaceae, Rikenellaceae*^26^. Hence, the intake of different structures on dietary polysaccharides affects the colonization of intestinal microorganisms^23^.

In addition, the intake of dietary polysaccharides alters the mucus layer, mucin, and its *O*-glycans. Supplementation with naturally extracted LBPs and raspberry pectin (PEC), commercially available konjac glucomannan (KGM), and inulin (INU) results in a significantly thicker mucus layer^26–29^. Muc2 secretion in the mouse ileum and colon is promoted by supplementation with natural extracts of *Dendrobium huoshanense* polysaccharides, commercially available carrageenan, chondroitin sulfate (CS), and sodium alginate^30,31^. Addition of NutriKane and Benefiber polysaccharides (Gratuk Technologies Pty. Ltd., Macquarie Park, NSW, Australia) is associated with increased levels of fucosylated *O*-glycans and decreased levels of sialylated *O*-glycans in colonic mucin of obese mice^12^. The effect of crude or single dietary polysaccharides on intestinal mucus layer thickness or mucin expression has been studied mainly in disease models. A systematic glycomics comparison in healthy mice could reveal the inherent effect of dietary polysaccharides on mucin *O*-glycans.

We hypothesized that different structural dietary polysaccharides might affect the profile of intestinal Muc2 *O*-glycans by influencing the composition of the intestinal community. To verify this hypothesis, the present study investigated the effect of supplementing structurally diverse dietary polysaccharides on the mucin *O*-glycan profile and gut microbial composition of healthy C57BL/6J mice. The mice were supplemented for 9 weeks with KGM, *Laminaria japonica* polysaccharides (BSP), CS, *L. barbarum* arabinogalactan (LBP-AG), PEC, INU, and a mixture of six polysaccharides (MIX). The relationship between dietary polysaccharide structure, mucin *O*-glycan profile, and gut microbiota was investigated. Our results can lead to the development of dietary polysaccharides for use as health-promoting supplements that stimulate the intestinal barrier function.

## Results

### Effect of structurally diverse dietary polysaccharides on mouse colon morphology

**Fig. 1:**
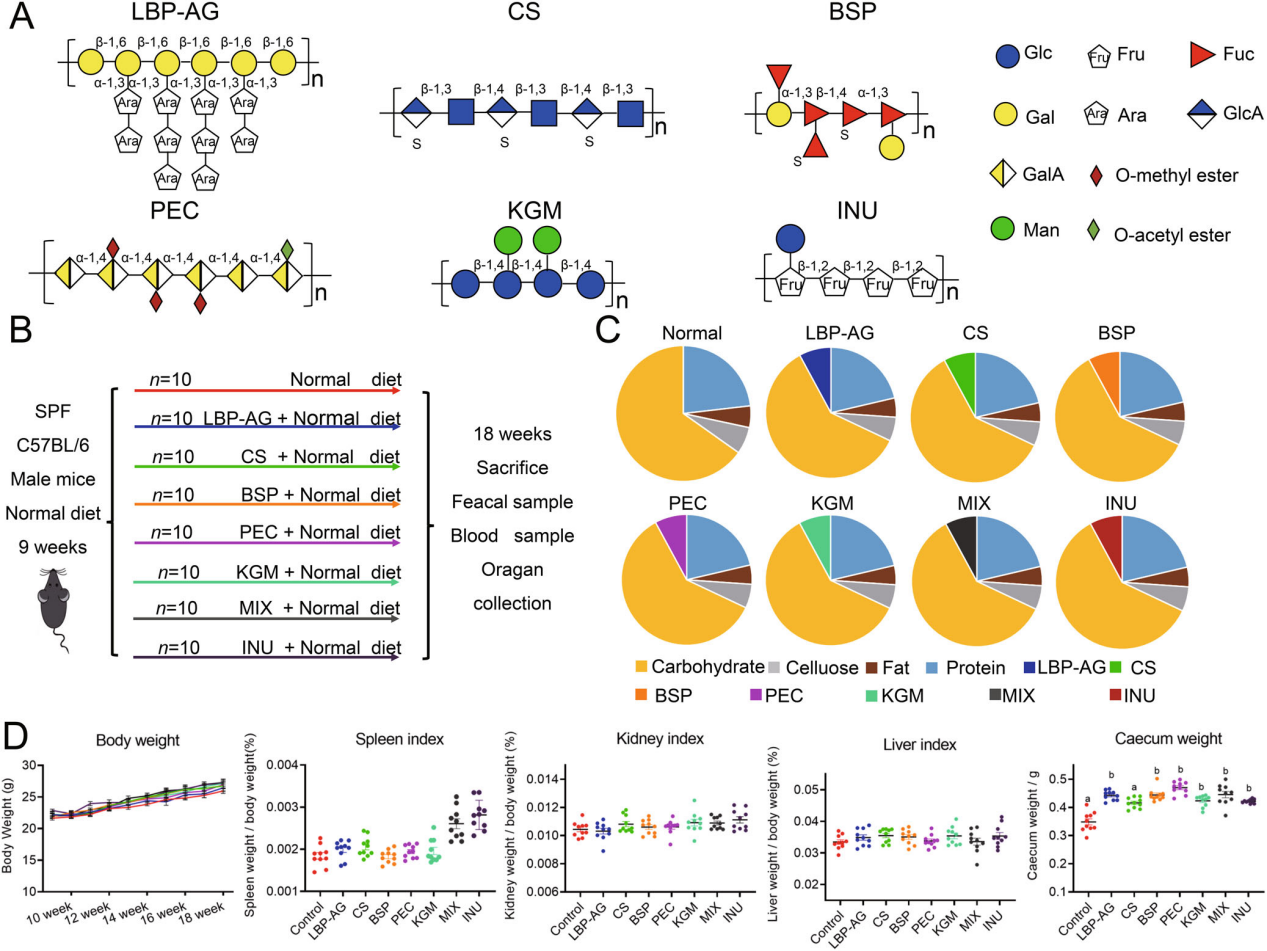
Structure of the six dietary polysaccharides used in this study, experimental design, and physiological parameters of the mice during the study. **A** Structure of the six dietary polysaccharides used. **B** Timeline of the experiment. The mice were fed a normal diet or a diet containing the different polysaccharides (*n* = 10) for 18 weeks. The time and type of samples collected, and measurements performed are indicated. **C** Nutrient composition of each diet. Detailed nutritional information is provided in Supplementary Table 2. **D** Weekly changes in body weight per mouse (mean ± SD); organ index for the spleen, kidneys, and liver (mean ± SEM); and the weight of the caecum (mean ± SEM). Significance (*P* < 0.05) was determined using Duncan’s test (*n* = 10).

To explore how structurally diverse dietary polysaccharides affected colonic mucus *O*-glycans, C57BL/6J mice were allocated randomly to eight groups and fed either a normal diet (CON) or a normal diet supplemented with different polysaccharides (LBP-AG, CS, BSP, PEC, KGM, MIX, and INU) as shown in Fig. 1A. A detailed timeline of the experiment and the proportions of major nutrients in the diet are illustrated in Fig. 1B, C. Physiological parameters, such as body weight, spleen index, kidney index, and liver index (Fig. 1D) displayed no significant change on a weekly basis (*P* > 0.05), indicating that polysaccharide supplementation had no adverse effect on host health. Supplementation with dietary polysaccharides increased cecum weight more than in CON group, albeit without any significant difference across dietary polysaccharide groups (Fig. 1D).

**Fig. 2:**
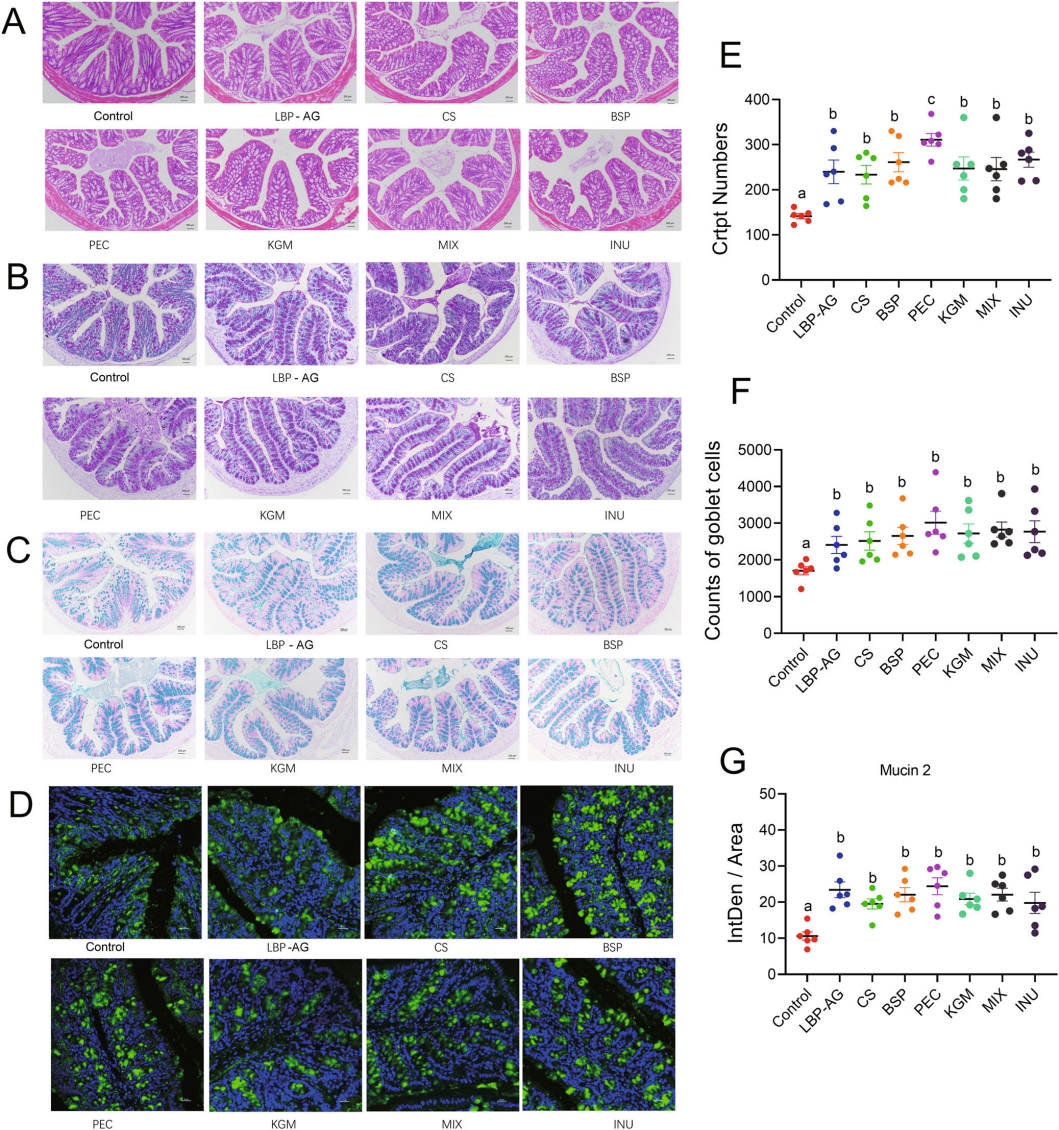
Histological and immunoreactive staining of distal colon tissue in mice from each treatment group, as well as corresponding quantification. **A** Hematoxylin and eosin staining (scale bars, 200 μm). **B** PAS staining (scale bars, 200 μm). **C** Alcian blue staining (scale bars, 200 μm). **D** Fluorescent immunostaining of mucin 2 (scale bars, 20 μm). **E** Crypt number (mean ± SEM). **F** Number of goblet cells (mean ± SEM). **G** Quantification of mucin 2 in the goblet cells (mean ± SEM). Significance (*P* < 0.05) was determined using Duncan’s test (*n* = 6).

To evaluate whether polysaccharide administration altered mucus morphology, colon tissues were stained with hematoxylin and eosin, alcian blue, as well as periodic acid-schiff (PAS) in Fig. 2A–C. Mucin 2 of goblet cells was detected by immunofluorescence staining (Fig. 2D). The number of crypts and goblet cells in the mouse colon was calculated by gray-scale analysis of PAS images, and was significantly higher in dietary polysaccharide-supplemented animals compared to the control group (Fig. 2E, F), along with higher fluorescent immunostaining of Muc2 in the colon of mice (Fig. 2G). Again, there was no significant difference among dietary polysaccharide groups. To investigate the effect of dietary polysaccharides on gut barrier function in mice, tight junction proteins, including ZO-1, occludin, and claudin, were semi-quantified by western blotting (Supplementary Fig. 1A–D). All dietary polysaccharides caused a significant increase in the expression of these proteins in the colon (*P* < 0.05). The effect was particularly pronounced in the MIX group, indicating that its rich monosaccharide composition could improve the intestinal barrier of mice more than other dietary polysaccharides.

### Supplementation with structurally diverse polysaccharides alters *O*-glycosylation of the colonic mucus layer

To determine the impact of structurally diverse polysaccharides on the profile of scraped colonic mucus layer *O*-glycans, the latter were released from equal amounts of intestinal mucin by the reductive β-elimination method. Equimolar amounts of β-cyclodextrin and 6’-sialyllactose were added as internal standards. Neutral and acidic *O*-glycans on Muc2 were analyzed in positive and negative ion mode, respectively. Relative quantification of colonic Muc2 *O*-glycans was performed using online tandem liquid chromatography (LC)-mass spectroscopy (MS). The structure of *O*-glycans was presumed based on MS (*m/z*) values, GlycoWorkbench version 2.1, and published reports^3,12,32^. Supplementary Table 3 lists total *O*-glycans, neutral and acidic *O*-glycans, non-fucosylated and fucosylated neutral *O*-glycans, sialylated and sulfated *O*-glycans, fucosylated *O*-glycans, both sulfated and sialylated *O*-glycans, as well as core 2, core 1, and core 4 glycans. Fifty-four types of *O*-glycans were identified in the CON group, with their LC-MS profile peaks reported in Supplementary Figs. 2 and 3, and the MS/MS profile of the isomer was shown in Supplementary Fig. 4. Even though the same *O*-glycans were identified in the CON and dietary polysaccharide groups, their relative levels differed significantly (*P* < 0.05).

**Fig. 3:**
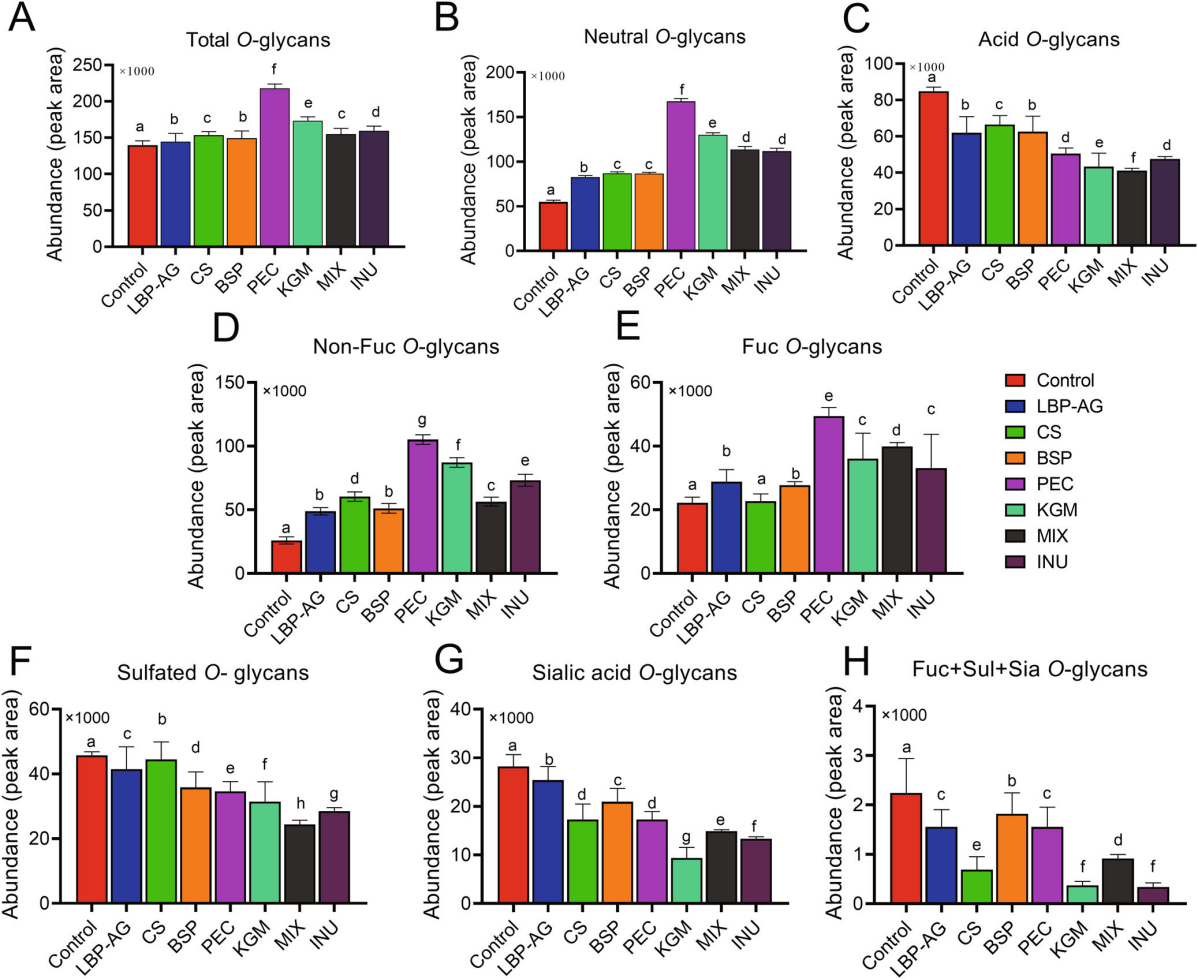
Characterization of *O*-glycans present in mouse colonic mucin. **A**–**C** Abundance of total (**A**), acidic (**B**), and neutral (**C**) *O*-glycans. (**D**–**H**) Subdivision according to specific modifications: non-fucosylated neutral *O*-glycans (**D**), fucosylated neutral *O*-glycans (**E**), sulfated *O*-glycans (**F**), sialylated *O*-glycans (**G**), and sulfated and sialylated acidic *O*-glycans (**H**). Data are presented as the mean ± SEM. Significance (*P* < 0.05) was determined using Duncan’s test (*n* = 5).

Monosaccharide composition, presumed structure, *m/z* values, abundance peaks, and number of colonic Muc2 *O*-glycans in all groups are summarized in Supplementary Table 5. *O*-glycosylation levels were determined by comparative analysis, which revealed that intestinal mucin *O*-glycans increased upon supplementation with dietary polysaccharides (Fig. 3A). The total amount of *O*-glycans was significantly higher (*P* < 0.05) in the PEC group compared to other groups and 1.71 times higher than in the CON group. Further analysis demonstrated a significant increase in neutral *O*-glycans, but a decrease in acidic *O*-glycans upon supplementation with dietary polysaccharides (Fig. 3B, C). Again, neutral *O*-glycans were significantly more abundant in the PEC than in other groups (*P* < 0.05), and 2.97-fold more abundant than in the CON group. Acidic *O*-glycans were particularly scarce in the MIX group, whereby their level was 0.55 times lower than in CON group.

**Fig. 4:**
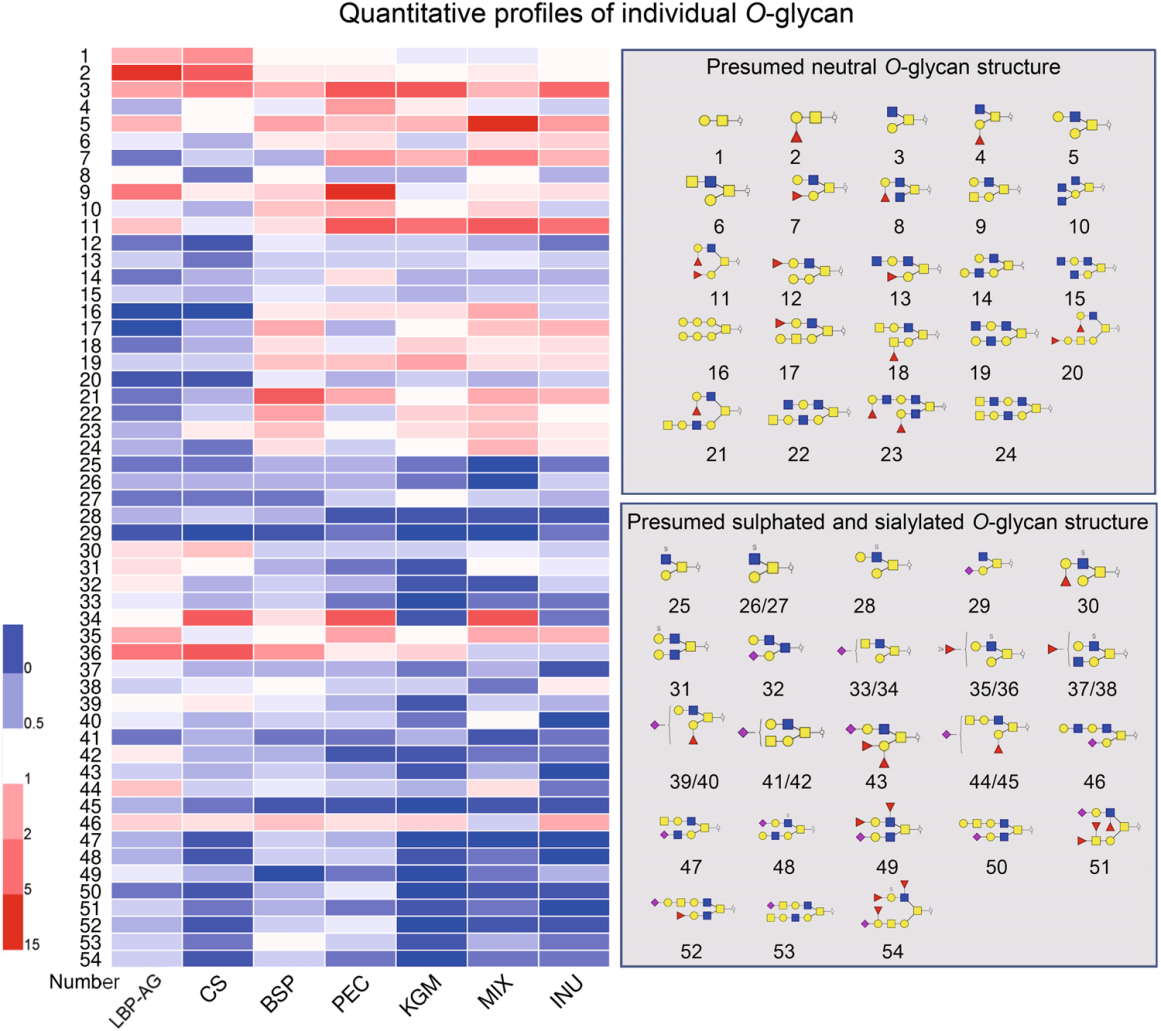
Quantitative profiles of individual *O*-glycans in each polysaccharides group and presumed structure of *O*-glycans. The ratios of *O*-glycans in LBP vs. CON, CS vs. CON, BSP vs. CON, PEC vs. CON, KGM vs. CON, MIX vs. CON, and INU vs. CON groups are shown on the left; the structure of *O*-glycans is reported on the right.

Neutral *O*-glycans were further assessed in terms of non-fucosylated and fucosylated glycans (Fig. 3D, E). Non-fucosylated *O*-glycans were more abundant upon supplementation with the different dietary polysaccharides, as shown in Fig. 4 for core 2 Gal-(GalNAc-6)GalNAcol (Number 3), whose content increased by ~2–4 fold. Compared to the CON group, the PEC group presented 14.73-fold higher levels of terminally non-fucosylated neutral *O*-glycans (H2N3, Number 9); whereas the MIX group led to 17.42-fold higher levels of core 2 *O*-glycans containing galactose (H2N2, Number 5). All dietary polysaccharide groups caused an increase in the content of terminally fucosylated neutral *O*-glycans, with the PEC and MIX groups achieving 2.24 times and 1.80 times higher levels, respectively, than the CON group (Fig. 3E). Fucosyltransferase 2 (Fut2), which mediates the addition of Fuc to Gal via an α-1-2 linkage, is the only fucose glycosyltransferase in mice^3^. The expression of Fut2 in the colon of mice was determined in different dietary polysaccharide groups (Supplementary Fig. 1A, E). Fut2 expression was similar to that of fucosylated *O*-glycans on mucin, with the PEC group again exhibiting significantly higher levels than other groups.

As shown in Fig. 4, the LBP group exhibited 13.13 times higher levels of the terminally mono-fucosylated core 1 *O*-glycan (Fucα1,2Galβ1,3GalNAcol, Number 2); PEC and MIX increased by 4.92 and 4.26 times the level of terminally di-fucosylated F2H2N2 (Number 11); and CS and PEC augmented by 1.19 and 2.51 times the amount of F1H1N2, another terminally fucosylated *O*-glycan (Number 2). Consistent with our results, supplementation with corn-resistant starch boosts the activity of fucosyltransferase in the small intestine of normal rats and, consequently, the amount of fucosylated *O-*glycans^33^.

Acidic *O*-glycans were further analyzed in terms of sulfated *O*-glycans, sialylated *O*-glycans, and both sulfated and sialylated *O*-glycans (Fig. 3F–H). All three *O*-glycan types were less abundant following dietary polysaccharide supplementation (*P* < 0.05). Furthermore, a decrease in *O*-glycans with a terminal sulfate group (S1H1N2, Number 25) and sulfated *O*-glycans with a terminal fucose (S1F1H1N2, Numbers 26 and 27) contributed to fewer sulfated *O*-glycans, as outlined in Figs. 3F–H and 4. Sulfated *O*-glycan content was much lower (46.5%) in the MIX group compared to all other groups (*P* < 0.05). Instead, sialylated *O*-glycans were particularly scarce (66.7%) in the KGM group, and included H2N3A1, H1N3A1, and another 10 glycoforms. Lower levels were detected also in the INU (52.7%) and MIX (47.3%) groups. The KGM and INU groups displayed a similar drop in sialylated *O*-glycans harboring a terminal fucose (F1H2N2A1, Numbers 39 and 40; F1H3N2A1, Numbers 44 and 45). For both sulfated and sialylated *O*-glycans, the INU and KGM groups presented comparable reductions of 85.2% and 83.4%, respectively, whereas the CS and MIX groups displayed 69.6% and 59.3% lower amounts, respectively. Overall, the MIX, INU, and KGM groups exhibited significantly (*P* < 0.05) lower levels of sulfated *O*-glycans, sialylated *O*-glycans, and both sulfated and sialylated *O*-glycans, respectively. Among acidic *O*-glycans, sulfated and sialylated *O*-glycans showed a particularly strong reduction in the MIX group; whereas the KGM group affected negatively sialylated *O*-glycans. The abundance of S1F2H2N2 (*m/z* = 1121) in colonic mucins of obese mice was reported to decrease following supplementation with insoluble NutriKane and Benefiber^12^. Here, instead, S1F2H2N2 showed an increase, which might be explained by differences in mouse models and type of fiber.

### Effect of dietary polysaccharides with different structures on gut microbiota

**Fig. 5:**
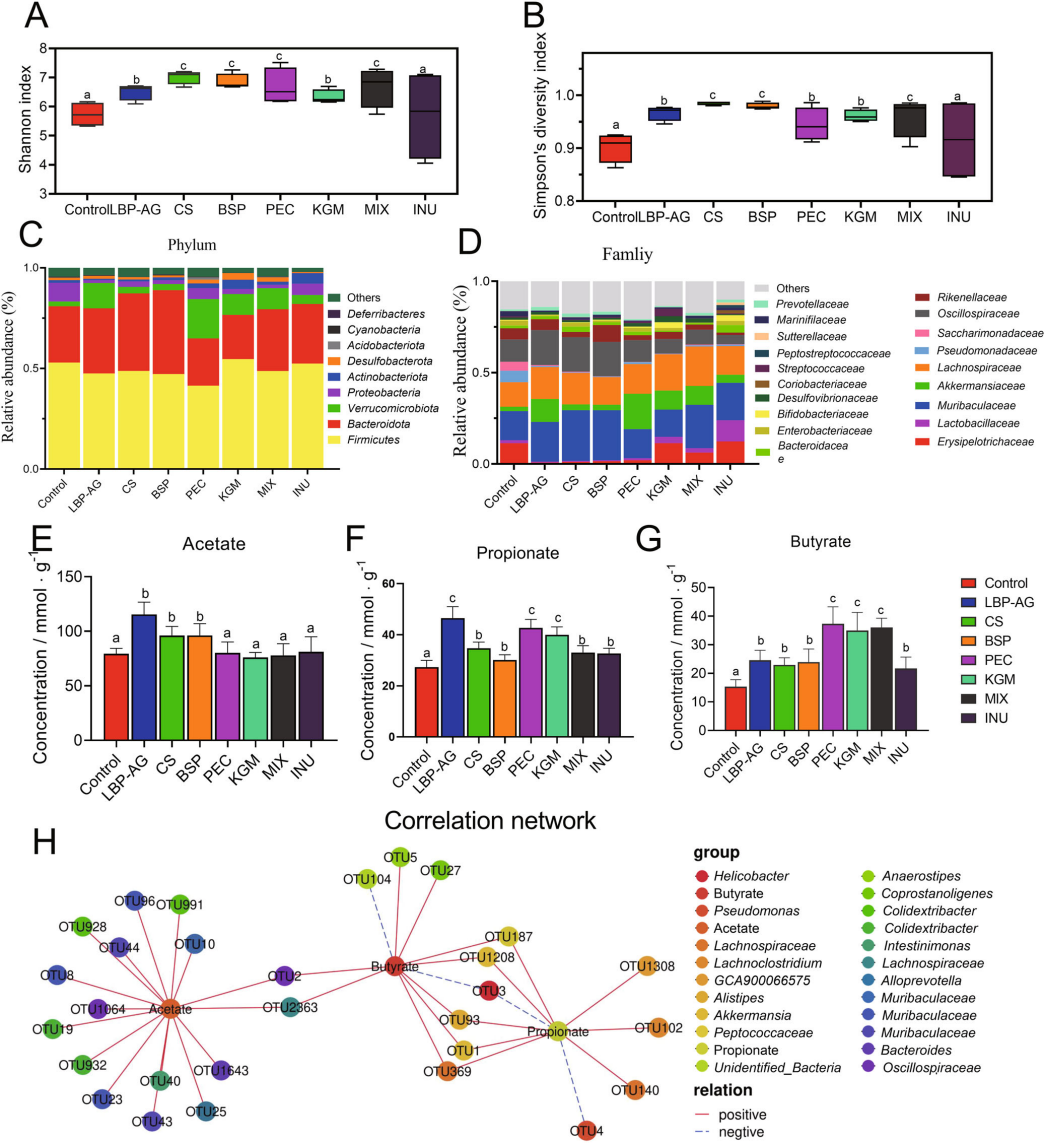
Diversity and relative abundance of gut microbiota, along with SCFA content in different dietary polysaccharide groups. **A**, **B** Diversity indices: Shannon index (**A**) and ACE index (**B**). **C**, **D** Relative abundance of bacteria at phylum (**C**) and family (**D**) level; only abundances >1% are shown. **E**–**G** SCFA content in the caecum (mean ± SD): acetic acid (**E**), propionic acid (**F**), and butyric acid (**G**). **H** Spearman correlation between SCFAs and gut bacteria. Significance (*P* < 0.05) was determined by Duncan’s test (*n* = 6).

The influence of structurally diverse polysaccharides on fecal gut microorganisms was demonstrated using 16S rRNA gene amplicon sequencing. The ordination of the gut microbiota in all groups was significant (*P* < 0.05) by principal component analysis (PCA) and Non-metric multidimensional scaling (nMDS plot) as shown in Supplementary Fig. 5 (includes additional analyses of β-diversity using PERMANOVA, Con vs. LBP: *P* = 0.019; Con vs. CS: *P* = 0.027; Con vs. BSP: *P* = 0.046; Con vs. PEC: *P* = 0.003; Con vs. KGM: *P* = 0.007; Con vs. MIX: *P* = 0.03; Con vs. INU: *P* = 0.03). Animals supplemented with PEC and MIX displayed higher ɑ-diversity and richness than the CON group or those fed other dietary polysaccharides (Fig. 5A, B). The major phyla were *Firmicutes*, *Bacteroidetes*, *Verrucomicrobiota*, and *Proteobacteria*, which together represented about 90% of taxa (Fig. 5C). Compared to the CON group, dietary polysaccharides significantly reduced the relative abundance of *Proteobacteria* (*P* < 0.05). The PEC group resulted in significantly more *Verrucomicrobiota*, which were also enriched in the MIX and LBP-AG groups along with *Bacteroidetes*. The latter were also abundant in the CS and BSP groups.

**Fig. 6:**
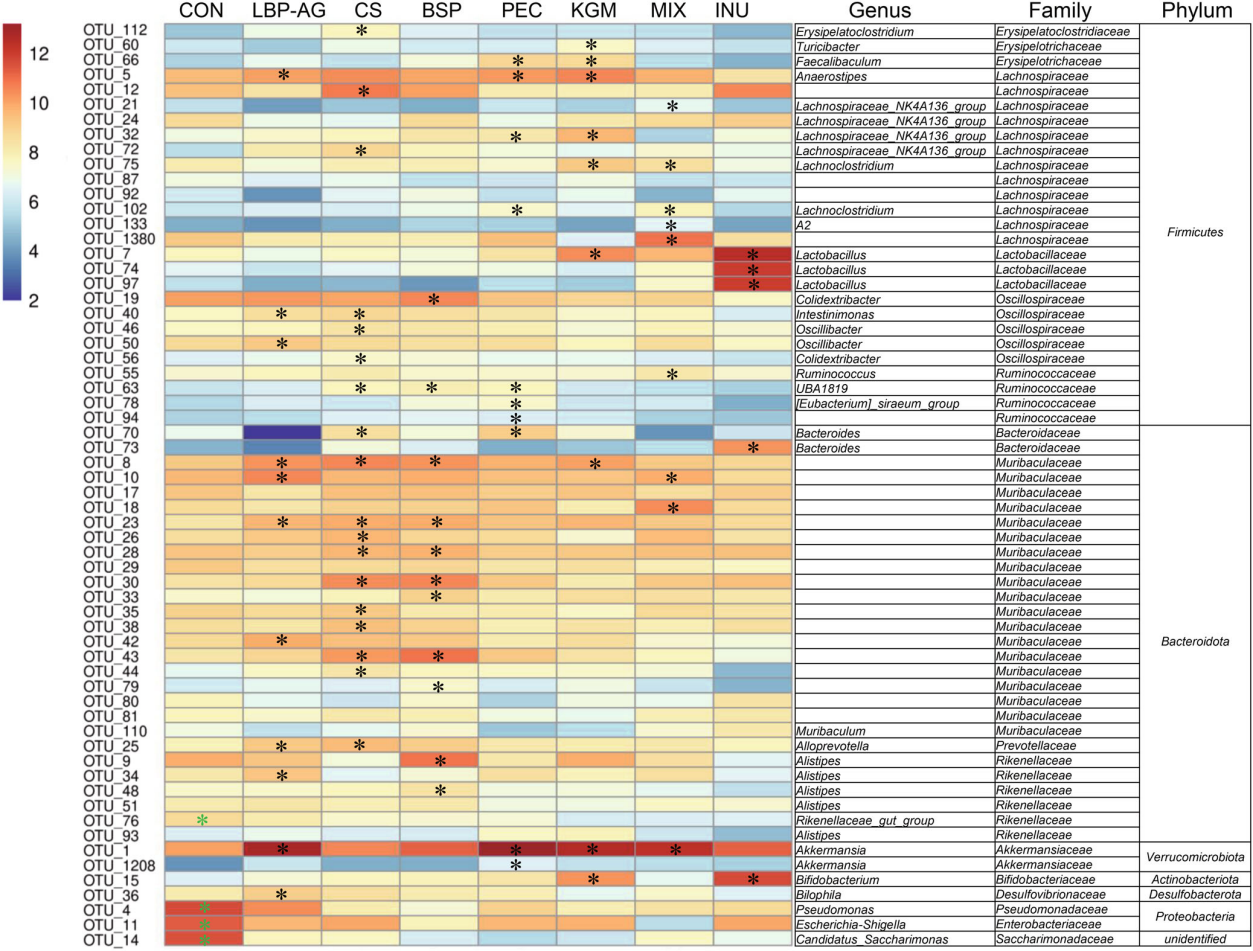
Relative abundance (Log_2_ transformed) of OTUs was significantly different between dietary groups. Differentially abundant OTUs were determined through LDA analyses (LDA > 3) between CON vs. LBP, CON vs. CS, CON vs. BSP, CON vs. PEC, CON vs. KGM, CON vs. MIX, and CON vs. INU (the LDA image and heatmap of relative abundance for each mouse were provided in Supplementary Figs. 6 and 7). The taxonomic level of OTUs (phylum, family, and species) is indicated on the right. Note: “*” (Black) Greater abundance in LBP, CS, BSP, PEC, KGM, MIX, and INU groups compared to CON; “*” (Green) Lower abundance in LBP, CS, BSP, PEC, KGM, MIX, and INU groups compared to CON.

Comparison at the family (Fig. 5D) and operational taxonomic unit (OTU) level (Fig. 6 and Supplementary Figs. 6 and 7) using a linear discriminant analysis (LDA) effect size (LEfSe) method (LDA > 3) revealed fewer *Pseudomonadaceae* (OTU 4) following supplementation with dietary polysaccharides. The MIX group yielded significantly (*P* < 0.05) more OTUs in the family *Lachnospiraceae* (OTUs 1380, 102, 75, 21, and 133) compared to the CON or other polysaccharide groups. The relative abundance of two OTUs of the genus *Akkermansia* (OTUs 1 and 1208) was significantly (*P* < 0.05) higher after supplementation with arabinose-rich PEC, LBP-AG, and MIX. This finding confirms a previous report, whereby a polysaccharide containing arabinose correlated positively with the relative abundance of *Akkermansia*^34^. OTUs of the *Muribaculaceae* were increased in the CS (OTUs 28, 8, 43, 30, 23, 44, 26, 35, 25, and 38) and BSP (OTUs 28, 8, 43, 30, 23, 33, 79, and 44) groups rich in sulfated polysaccharides (Fig. 6). Three OTUs in the genera *Bifidobacterium* (OTU 15) and *Lactobacillus* (OTUs 7 and 74) were higher in the KGM and INU groups. The prebiotic INU was previously reported to improve the relative abundance of *Lactobacillus* and *Bifidobacterium*^7,28^, which is consistent with our results. Overall, the various dietary polysaccharides had specific effects on intestinal microbial taxa. Besides OTUs, the contents of butyrate, propionate, and acetate were also assessed in the different groups of mice (Fig. 5E–G). The PEC and MIX groups significantly (*P* < 0.05) enhanced butyrate content, which was 2.43 times and 2.35 times higher than in the CON group, respectively. Propionate and acetate levels were 1.72 times and 1.45 times greater in the LBP-AG than in the CON group, respectively. Spearman correlation revealed a significant association between SCFA content and specific intestinal bacterial OTUs (Fig. 5H and Supplementary Table 4). The relative abundance of *Akkermansia* (OTUs 1 and 1208), *Lachnospiraceae_NK4A136_group* (OTU 2363), and *Peptococcaceae* (OTU 187) correlated positively with the concentration of butyric acid. Therefore, the increase in butyric acid content in the PEC and MIX groups might be related to enrichment with *Akkermansia* and *Lachnospiraceae_NK4A136_group*. Indeed, *Lachnospiraceae_NK4A136_group* has been shown to promote the release of butyrate^35^. Propionate content was positively related to the abundance of OTUs in the genera *Lachnoclostridium* (OTU 102) and *GCA-900066575* (OTU 1308).

**Fig. 7:**
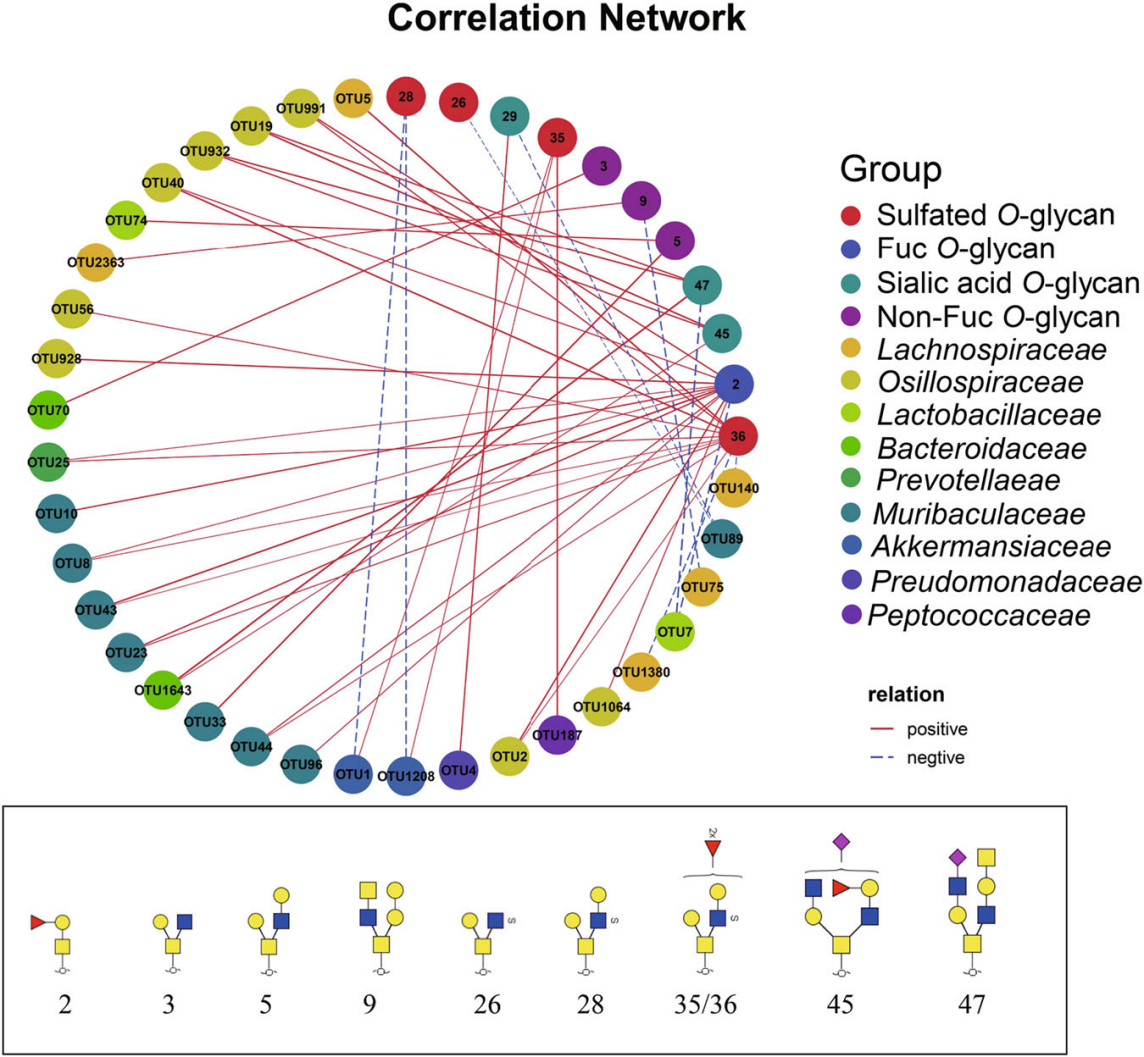
Network revealing correlations between mouse colonic mucin *O*-glycans and bacterial OTUs. Values are based on pairwise Spearman correlation between the number of *O*-glycans and the relative abundances of OTUs. Only *O*-glycans and OTUs with significantly different abundances are displayed. Significant correlations (*P* < 0.05) used to construct the network are shown.

Spearman correlation revealed the relationship between *O*-glycans and bacterial OTUs (Fig. 7). The relative abundance of OTUs within the genus *Bacteroides* (OTU 70) correlated positively with the content of non-fucosylated *O*-glycans such as H1N2 (Number 3). Enrichment of OTUs from *Lactobacillus* (OTU 74) and *Muribaculaceae* (OTU 3) correlated positively with the abundance of the H2N2 glycoform (Number 5). F1H1N1 (Number 2) correlated positively with OTUs in the families *Prevotellaceae* (OTU 25), *Muribaculaceae* (OTUs 10, 8, 43, 23, 44, and 96), and *Oscillospiraceae* (OTUs 991, 40, 2, 1064, and 928); but negatively with OTUs in the *Lactobacillaceae* (OTU 7). *Parabacteroides*, *Lachnoclostridium*, and other bacteria stimulate the expression of α-1,2 fucosyltransferase in healthy human feces^36^, thereby promoting the production of fucosylated *O*-glycans on intestinal mucin, although this is not observed in patients with colitis^37^. The difference in microbial composition between this and previous studies may be related to distinct gut microorganisms in human vs. mouse feces. Furthermore, sulfated *O*-glycan structures such as S1H2N2 (Number 26) correlated negatively with the relative abundance of OTUs in the genus *Akkermansia*. Instead, S1F2H2N2 (Number 36) *O*-glycans correlated positively with the relative abundance of OTUs in *Lachnospiraceae* (OTU 5), *Muribaculaceae* (OTUs 43, 8, 23, and 44), and *Oscillospiraceae* (OTUs 991, 40, 56, and 2). Acidic *O*-glycans containing Neu5Ac are more abundant in the proximal part of the mouse colon, whereas fucosylated *O*-glycans tend to be more abundant in the distal colon, this distribution contrasts with that in humans, whereby fucosylated *O*-glycans predominate in the proximal gastrointestinal tract and more acidic *O*-glycans in the distal one^38^. In line with our results, sulfated and sialylated *O*-glycans in mouse colonic Muc2 were reported to correlate positively with OTUs in *Muribaculaceae, Lachnospiraceae*, and *Clostridiales*^12^. In addition, sialylated *O*-glycans F1H3N2A1 and H2N4A1 exhibited a positive correlation with OTUs within the families *Oscillospiraceae* (OTUs 19 and 932) and *Bacteroidaceae* (OTU 1643).

## Discussion

In this study, 54 *O*-glycans were detected in colonic Muc2 of healthy mice. This value exceeds the 37 or 36 *O*-glycans reported previously^12^, and might be explained by the separate detection of neutral and acidic *O*-glycans using positive and negative ion modes to improve MS sensitivity. Tardy et al.^39^ reported that dietary polysaccharides significantly enhanced the abundance of total and neutral *O*-glycans on Muc2 in the large intestine of rats by gas chromatography (GC) analysis of glycoprotein sugars. Our results demonstrate that dietary polysaccharides increased the amount of total Muc2 *O*-glycans and neutral *O*-glycans using LC-MS, which could be ascribed mainly to an increase in non-fucosylated *O*-glycans.

Both the PEC and MIX groups significantly increased the expression of neutral fucosylated *O*-glycans in mouse colonic mucin; whereas the MIX group significantly decreased the content of sulfated and sialylated *O*-glycans. This disparity might be related to monosaccharide composition and the abundance of glycosidic bonds. The MIX group presented a richer monosaccharide composition and greater glycosidic bond variation than the acidic PEC group, in which the predominant species was α-1,4 galacturonic acid. Hence, the modulatory effect of dietary polysaccharides on *O*-glycans was related to monosaccharide and glycosidic bond heterogeneity. To this end, corn-resistant starch (containing α-1,4 glucose linkages) has been shown to significantly boost the level of fucosylated *O*-glycans^39^. PEC is characterized by the same α-1,4 linkages, suggesting that the presence of fucosylated *O*-glycans on mucin might be affected by α-1,4 glycosidic linkages. LBP, a highly branched structure characterized by a main chain of β-1,6-Gal units and mostly arabinose substitutions at the O-3 position, significantly increased the content of terminally mono-fucosylated core 1 *O*-glycans. PEC, a linear heteropolysaccharide harboring α-1,4 glycosidic linkages in the main chain, KGM, a linear glucomannan with β-1,4 linkages, and INU, a linear oligofructose with α-1,2 linkages, boosted the levels of di-fucosylated *O*-glycans. Therefore, highly branched dietary polysaccharides appear to stimulate mono-fucosylated core 1 *O*-glycan chains; whereas linear dietary polysaccharides seem to favor di-fucosylated core 2 *O*-glycan chains. CS, which consists of a repeating disaccharide unit composed of β-1,3-linked glucuronide and GalNAc, as well as PEC, significantly decreased the level of mono-fucosylated *O*-glycans such as F1H1N2. This result indicates that glyoxylate-based dietary polysaccharides could specifically induce terminally mono-fucosylated core 2 *O*-glycans. In general, supplementation with PEC and MIX significantly enhanced the content of fucosylated *O*-glycans, but MIX elicited a stronger reduction in sulfated and sialylated *O-*glycans. Accordingly, supplementation with a varied mixture of monosaccharides containing different glycosidic bonds had a synergistic and more beneficial effect compared to that triggered by single dietary polysaccharides.

Colonization by specific intestinal bacteria influences *O*-glycosylation of mucin. For example, oral administration of the probiotic *L. farciminis* significantly reduced the expression of intestinal sialylated *O*-glycans (*m/z* 1331 and 1605) in irritable bowel syndrome^18^. Furthermore, intestinal bacteria affect glycosyltransferase activity, which in turn alters the degree of *O*-glycosylation on mucin. Our results demonstrate that differently structured dietary polysaccharides could regulate specific intestinal bacteria. PEC caused a significant rise in the relative abundance of *Bacteroides*, *Lachnospiraceae*, and *A. muciniphila*, following a shift from neutral non-fucosylated *O*-glycans to H2N2 and F2H2N2. LBP-AG boosted the relative abundance of *Prevotellaceae, Muribaculaceae, Oscillospiraceae*, and *A. muciniphila* by favoring mono-fucosylated neutral *O*-glycans such as F1H1N1. KGM promoted OTUs associated with *Bifidobacterium, Lactobacillus*, and *Muribaculaceae*, while decreasing the level of sialylated *O*-glycans such as F1H3N2A1. In addition, Spearman analysis revealed positive correlations between non-fucosylated *O*-glycans (H1N2 and H2N2, Numbers 3 and 5) and OTUs from *Bacteroides, Lactobacillus*, and *Muribaculaceae*; as well as between the mono-fucosylated *O*-glycan F1H1N1 (Number 2) and OTUs from the *Prevotellaceae, Muribaculaceae, Oscillospiraceae, Lachnospiraceae*, and *Lactobacillaceae* families. In contrast, negative correlations were found between sulfated *O*-glycans (S1H2N2 and S1F2H2N2, Numbers 26 and 36) and OTUs associated with *A. muciniphila*, *Lachnospiraceae, Muribaculaceae*, and *Oscillospiraceae*; as well as between sialylated *O*-glycans (F1H3N2A1 and H2N4A1, Numbers 45 and 47) and OTUs in the *Oscillospiraceae* and *Bacteroidaceae* families. Therefore, differentially structured dietary polysaccharides might regulate the expression of mouse colonic mucin *O*-glycans by affecting the composition of intestinal bacteria, and vice versa.

Free monosaccharides/oligosaccharides or SCFAs produced by fermentation of dietary polysaccharides indirectly alter the *O*-glycosylation pattern of intestinal mucins^17,22,23^. *Bacteroides* not only increase the expression of Fut2 in mouse intestinal colonic goblet cells, but they also generate a variety of dietary polysaccharide hydrolases, such as GH2, GH35, and GH42. These, in turn, could degrade mono- and oligosaccharides, including Gal, rhamnose, and arabinose, in the pectin structure and release them into the intestine^23^. Free Gal might be converted to UDP-Gal via the Leloir pathway to be used as a substrate for the glycosylation of the α1,2Galβ1,3GalNAcol intestinal mucin *O*-glycan core 2 structure^40^. GH3, GH43, GH13, GH2, and GH36 hydrolases produced by the *Lachnospiraceae* family metabolize butyrate and have been shown to improve colonic mucin^16,41,42^. The latter was found to increase the relative abundance of *A. muciniphila*, which used mucin *O*-glycans as a carbon source^43^. Our results revealed that the intestinal bacteria of mice in the PEC group were dominated by *Bacteroides*, *Lachnospiraceae_NK4A136_group*, and *A. muciniphila*. The same group was characterized by significantly higher levels of total *O*-glycans and neutral fucosylated *O*-glycans, which might be attributed to upregulation Fut2 expression due to an increased abundance of *Bacteroides-*related OTUs. Meanwhile, PEC could also enhance the expression of *O*-glycans through interactions of *Lachnospiraceae* and *A. muciniphila*. *Prevotellaceae* encodes specific trsusC/D polysaccharide utilization sites that produce hydrolases (e.g., GH137 and xynD)^44^, degrade dietary polysaccharides into free monosaccharide or oligosaccharide units, and release acetate, which promotes KLF4 signaling and consequently inhibits neutral *O*-glycans in mouse colonic goblet cells^17^. Our results point to a significantly higher relative abundance of *Prevotellaceae* and acetate content in the LBP-AG, CS, and BSP groups compared to other dietary polysaccharide groups; but a lower content of neutral *O*-glycans. We hypothesize that this observation might be due to LBP-AG, CS, and BSP being able to increase acetate levels by stimulating the growth of *Prevotellaceae* and consequent downregulation of neutral *O*-glycan chains in mouse intestinal mucins. The *O*-glycan profile was similar for INU (composed of β-1,2-linked fructose) and KGM (composed of glucomannan linked by β-1,4 glycosidic bonds), and was characterized by fewer terminally mono-fucosylated and sialylated *O*-glycans. This similarity might be explained by the degradation of free mannose by intestinal microorganisms (*Bifidobacterium* and *Lactobacillus*)^45^, as well as its isomerization to fructose via phosphomannose isomerase. The impact of structurally diverse dietary polysaccharides on intestinal bacteria or colonization with specific intestinal microorganisms on the *O*-glycans of intestinal mucins has been reported previously^18,23^.

In this study, we investigated changes to the intestinal microbial composition and its metabolites (e.g., SCFAs), as well as the *O*-glycans of colonic mucins following the administration of six structurally defined dietary polysaccharides. The correlation between intestinal microbes and colonic mucin *O*-glycans was analyzed using bioinformatics, and the possible relationship between dietary polysaccharide structure, intestinal flora, and colonic mucin *O*-glycans was explored. These correlations could help clarify the relationship between colonic mucin *O*-glycans and intestinal microbiota in both healthy and diseased individuals.

In summary, dietary polysaccharides maintain a healthy gut by influencing the colonic mucus layer of highly *O*-glycosylated mucins. This is the first study to elucidate how intestinal mucin *O*-glycosylation is influenced by polysaccharide structure. Highly branched arabinogalactan favored terminally fucosylated core 1 *O*-glycans; whereas linear chain polysaccharides, including pectin, konjac glucomannan, oligofructose (inulin), and a polysaccharide mixture, increased significantly the abundance of terminally di-fucosylated *O*-glycans. In addition, the relationship between dietary polysaccharide structure, mucin *O*-glycan composition, and intestinal microorganisms was systematically compared, opening the door for targeted nutritional interventions.

## Methods

### Experimental model and subject details

#### Animal model

C57BL/6J mice, aged 6–8 weeks and weighing approximately 20.0–22.0 g, were housed under specific pathogen-free conditions. Standard lighting (12-h light/dark cycle), humidity, and temperature (25 ± 2 °C), as well as adequate food and germ-free drinking water (autoclaved) were provided. Animals were randomly assigned to eight groups, with 10 mice per group, after adaptive rearing for a week (Fig. 1B). Daily intake of the main nutrients is reported in Fig. 1C, and diet composition is reported in Supplementary Table 2. The different polysaccharides (LBP, CS, BSP, PEC, KGM, INU and their mixture) were dissolved in saline (0.9% NaCl), and gavaged daily (200 mg/kg/day) to mice for 9 weeks, the mixture group (MIX, 200 mg) was prepared six polysaccharide mixture with an equal amount; meanwhile, the CON group was orally administrated with an equal volume of saline. Body weight and feed quantity were recorded daily. During the last 3 days of the trial, each mouse was placed in the metabolism cage (LAT- XSDXL, Lab Animal Technology Develop Co., Beijing, China), respectively, and fecal samples were collected into sterile centrifuge tubes (1.5 mL) using tweezers and fast-frozen in liquid nitrogen for 16S rRNA sequencing. The mice were euthanized with isoflurane after 9 weeks of the dietary intervention, at which point their blood, cecal contents, cecum, colon, kidney, spleen, and liver were collected, weighted, and stored at –80 °C. All animal studies (including the mice euthanasia procedure) were approved by Experimental Animal Management and ethics guidelines of Northwest University in China (NWU-AWC-20210401M). The approval declaration is provided in Supplemental Information.

#### Polysaccharide extraction and determination of basic indices

For LBP-AG extraction, the fruits were crushed and extracted twice at 80 °C for 2 h, with the two resulting supernatants pooled together and concentrated at <45 °C. Next, the concentrated supernatants were precipitated for 12 h using ethanol, and the precipitates were dissolved in Sevage reagent (1:4 n-butanol:chloroform) to remove proteins. Finally, the samples were dialyzed for 3 days, concentrated, and precipitated with a graded ethanol series (30%, 50%, and 70%). The 70% precipitates were vacuum freeze-dried to obtain LBP-AG. KGM and CS were purchased from Hebei Bailingway Superfine Materials Co Ltd (Hebei, China), INU was bought from Aladdin (Shanghai, China), while BSP and PEC were obtained from Meiyichen Biotechnology Company (Jiangsu, China). KGM, CS, INU, BSP, and PEC were dialyzed (3000 kDa) and freeze-dried. Sugar content was detected with the phenol sulfuric acid method. Protein content was measured with a BCA kit (Beyotime Biotechnology, Nanjing, China). The monosaccharide components and molecular weight of different polysaccharides were measured as reported previously^26^. Protein and sugar content, monosaccharide composition, and molecular weights of polysaccharides are listed in Supplementary Table 1.

#### Colon pathological sections

Colons were rinsed with pre-chilled phosphate-buffered saline and were fixed using Carnoy’s fixative (*n* = 6). The colon tissue specimens were embedded in paraffin after dehydration with ethanol, sliced into sections of 2–3 µm, deparaffinized, and treated with periodic acid alcohol solution and Schiff reagent, as well as hematoxylin and eosin. After that, the samples were dehydrated and sealed. In addition, sections were placed in Alcian Blue Stain Solution A for 15 min, washed with water, stained with Alcian Blue Stain Solution B for 3 min, dehydrated, and sealed. Histological sections were photographed at ×4, ×10, and ×20 magnification using an inverted fluorescence microscope (ECLIPSE Ti2-A; Nikon, Tokyo, Japan). For magnifications above ×10, three random fields of view were imaged. In addition, the number of goblet cells and crypts (×4 magnification PAS) was determined using ImageJ version 1.51j8 (NIH, Bethesda, MD, USA).

#### Immunofluorescence staining of colonic mucin 2

The colon (1.0 cm) was fixed using Carnoy’s fixative (*n* = 6). The samples were dehydrated in ethanol, embedded in paraffin, sliced into sections of 2–3 µm, and incubated in the dark with primary antibodies against mucin 2 (1:2000, 27675-1-AP; Proteintech, Wuhan, China) and secondary antibodies (1:3000, G1215-200T; Servicebio, Wuhan, China). Finally, the slides were observed under a confocal microscope (AX-R; Nikon) at ×10, ×20, and ×40 magnification. Different fields of view were selected at random and imaged three times. Mucin 2 (×10 magnification) levels were quantified using ImageJ.

#### Western blotting

Mouse colon samples of 70–90 mg were weighted and minced (*n* = 6), before resuspending the resulting lysate in RIPA buffer (PR20001; Proteintech) containing phenylmethylsulfonyl fluoride (PMSF, 20016; Proteintech) as protease inhibitor. The samples were disrupted with an ultrasonicator and the supernatant was collected by centrifugation. Total protein content was assayed with the BCA method (Beyotime Biotechnology). Immunoblotting of β-actin (1:2000, GB1500; Servicebio), GADPH (1:5000, M20050S, Abmart, Shanghai, China), ZO-1 (1:500, GB11140; Servicebio), (1:1000, bs-12258R; Bioss, Beijing, China) occludin (1:500, GB111401, Servicebio), claudin 1 (1:500, GB11254; Servicebio), and Fut2 (1:1000, bs-12258R; Bioss, Beijing, China) was performed as described previously using a fluorescence chemiluminescence imaging system^26^. Images were processed with ImageJ, with each group assessed at least three times (*n* ≥ 4).

#### Sequencing and bioinformatics analysis of 16S rRNA gene amplicons

Briefly, 2–4 g (*n* = 6) of mouse feces were weighed and total genomic DNA of mouse fecal bacteria was extracted using a QIAamp DNA Stool Mini Kit (51504; QIAGEN, Holden, Germany).

Amplicons were generated as summarized hereafter. Different regions of the 16S rRNA gene (V3-V4) were amplified using specific primers with barcodes (319F/806R). All PCR reactions were performed with 15 μL Phusion® High Fidelity PCR Master Mix (New England Biolabs, Ipswich, MA, USA), 0.2 μM forward and reverse primers, and approximately 10 ng template DNA. Thermal cycling included initial denaturation at 98 °C for 1 min, followed by 30 cycles of denaturation at 98 °C for 10 s, annealing at 50 °C for 30 s, and extension at 72 °C for 30 s, plus a final step of 5 min at 72 °C.

To quantify and identify the PCR products, the same volume of SYBR (1×) loading buffer (containing SYBR Green) was mixed with the PCR products, which were then separated on a 2% agarose gel and purified with the Qiagen Gel Extraction Kit.

Sequencing libraries were generated using TruSeq® DNA PCR-Free sample preparation kits (Illumina, San Diego, CA, USA) according to the manufacturer’s recommendations and index codes were added. The libraries were quantified on a Qubit@2.0 Fluorometer (Thermo Scientific) and evaluated on an Agilent Bioanalyzer 2100 system (Agilent Technologies, Santa Clara, CA, USA). Finally, libraries were sequenced on the Illumina NovaSeq 6000 platform to generate 250-bp paired-end reads.

Paired reads were assigned to samples based on their unique barcodes, and truncated by cutting off barcodes and primer sequences. They were then combined using FLASH (VI.2.7, http://ccb.jhu.edu/software/FLASH/)^46^, a very fast and accurate tool designed to merge paired-end reads when at least some of them overlap with reads generated at the other end of the same DNA fragment. The spliced sequence, referred to as the original tag, was quality-filtered under specific filtering conditions following the QIIME V1.9.1 (http://qiime.org/scripts/split_libraries_fastq.html) quality control process to obtain high-quality clean labels^47,48^. The tags were compared to the reference Silva database (https://www.arb-silva.de/) using the UCHIME algorithm (http://www.drive5.com/usearch/manual/uchime_algo.html) to detect and remove chimeric sequences from the final label^49,50^. An average of 81,255 tags was measured per sample by splicing the reads, and an average of 80,637 valid data were obtained through quality control, yielding 62,022 valid data and a quality control efficiency of 76.35%. Sequences were clustered into OTUs with 97% identity using Uparse, which yielded a total of 2588 OTUs. Finally, the OTUs were annotated with the Silva138 database for species.

Non-metric multidimensional scaling (nMDS) plots and principal component analysis (PCA) were constructed based on Bray–Curtis similarity matrices of Log (*x* + 1) transformed abundance of the OTUs, Permutational Multivariate Analysis of Variance (PERMANOVA) tests based on Bray–Curtis distance measures to investigate differences in the microbial community structure, and PERMANOVA were analyzed by Prizer 7.0. Distinct phylotypes (families and OTUs) between dietary groups were identified using the LEfSe method with the following parameters: Kruskal-Wallis test among groups (*P* < 0.05), Wilcoxon test between groups (*P* < 0.05), and the threshold on the LDA score for discriminative features >3.0^51^. The correlation was determined using Spearman analysis. Bioinformatics analysis and image was performed using the OmicStudio tools at https://www.omicstudio.cn/tool.

#### Quantification of SCFAs

SCFA standards were prepared by transferring 9.52 μL acetic acid, 10.10 μL propionic acid, 10.10 μL butyric acid, 10.54 μL isobutyric acid, 10.66 μL n-valeric acid, 10.80 μL isovaleric acid, and 99.38 μL ether to a 15-mL centrifuge tube, which was then vortexed and mixed to obtain a stock solution of the six SCFAs. The stock solution was diluted to different concentrations (5, 50, 100, 200, 300, 400, and 500 mM).

Next, 100 mg of feces were transferred to a 2-mL centrifuge tube, followed by addition of steel beads for stirring, acidification with H_2_SO_4_ (50%, v/v), addition of diethyl ether, and vortexing twice. The fecal samples were ultrasonicated for 30 min in an ice bath at –20 °C, mixed with anhydrous sodium sulfate, vortexed, and finally centrifuged at 12,000 × *g* (15 min, 4 °C). The supernatant was filtered using a 0.22-μm organic filter.

Gas chromatography was carried out on a GC2010-PLUS instrument (Shimadzu, Kyoto, Japan), equipped with an HP-INNOWax column (0.25 μm, 0.250 mm × 30 mm; Agilent Technologies). The following parameters were used: inlet temperature, 250 °C; FID temperature, 230 °C; and split ratio, 10:1. The samples were heated to an initial temperature of 100 °C, ramped to 180 °C at 5 °C/min, and then held at 180 °C for 4 min. The separation took a total of 21 min, with a makeup of 9 min using air at 400 mL/min, N_2_ at 3 mL/min, and H_2_ at 40 mL/min.

#### Mucin sample collection and *O*-glycan structure determination

The colon was dissected, scraped with tweezers, and dissolved in 6 M guanidine chloride containing 0.01 M NaH_2_PO_4_ and 5 mM EDTA. PMSF (2 mM) was added prior to extraction with a glass mini grinder. After stirring with a pestle at 4 °C overnight, 10 mL of samples were reduced with dithiothreitol and methylate/iodoacetamide, dialyzed with 2 L of distilled water at 4 °C for 3 days (10 kDa), and changed distilled water twice a day at 8:00 am and 8:00 pm, transferred to centrifuge tubes, and freeze-dried.

*O*-glycans were released adding 1 mL NaOH (0.1 M) and 1 mL NaBH_4_ (1 M), and incubating for 15 h at 50 °C. β-Cyclodextrin (99.99%) and 6′-sialyllactose (99.9%) were added as internal standards, while acetate was added to adjust the pH to 4.5. The samples were purified using Solid-phase extraction (SPE) of C18 cartridges (200 mg/3 mL, SAC-C1803200; Simon Aldrich, Germany) and graphitic carbon SPE (200 mg/3 mL, Supelco; Bellefonte, PA, USA), A DEAE column (2 mg/3 mL; Biobomei, Hefei, China) was used to separate neutral and acidic *O*-glycans, which were detected under positive and negative ion modes, respectively (Supplementary Figs. 2 and 3).

*O*-glycans were analyzed using LC-MS. High-performance liquid chromatography was performed on a 1290 Infinity system (Agilent Technologies) using a Hypercarb column (3 μm, 2.1 mm × 100 mm; Thermo Scientific). Sample injection volume was 5 μL, column temperature was 60 °C, and flow rate was 0.450 mL/min. Mobile phases A (water) and B (1% acetonitrile in 0.1% v/v formic acid) were used to form the gradients as follows: 0–5 min, 100–85% A; 5–15 min, 85–70% A; 15–30 min, 70–60% A; 30–35 min, 60–30% A; 35–40 min, 30–5% A; 40–42 min, 5–2% A; 42–45 min, 2–0% A; and 45–50 min, 0–100% A. In negative ion mode, the program was as follows: 0–8 min, 100–85% A; 8–15 min, 85–75% A; 15–25 min, 75–70% A; 25–35 min, 70–60% A; 35–45 min, 60–40% A; 45–50 min, 40–2% A; 50–60 min, 2–0% A; and 60–65 min, 0–100% A.

LC-MS was carried out on a G6400 quadrupole time-of-flight mass spectrometer (Agilent Technologies) in positive or negative ion modes using a jet stream electrospray ionization source. The source parameters were as follows: sheath gas (N_2_), 400 °C at 12 L/min; drying gas (N_2_), 300 °C at 10 L/min; nozzle voltage, 1000 V; capillary voltage, 4750 V; fragment voltage, 175 V; and nebulizer pressure, 45 psi.

LC-MS data acquisition and analysis were performed using MassHunter Workstation software (Agilent Technologies) and Data Acquisition Workstation (v B.06.01, SP1). Peak areas and retention times were processed further in Microsoft Excel. Glycan compositions and structures were presumed according to a combination of the *m/z* value, regularity of *O*-glycans synthesis, published literature, and GlycoWorkbench version 2.1^3,12,32^.

### Statistical analysis

Statistical analyses were performed using one-way ANOVA with Duncan’s test by GraphPad Prism version 8.0 (GraphPad Inc., La Jolla, CA, USA) unless otherwise stated. For comparisons between two groups, an unpaired *t*-test was used when the samples were normally distributed. In all figures, data are presented as the mean ± standard error of the mean (SEM) or mean ± standard deviation (SD). Spearman correlations were used for data that were not normally distributed.

### Supplementary information

Supplementary-1

## Data Availability

All data supporting the findings of this study are available within the article and its Supplemental Information files. The non-curated MS raw data (MS/MS of isomer) was updated in UniCarbDR (https://unicarb-dr.glycosmos.org/references). The data of 16S rRNA gene amplicons are available from the NCBI under accession PRJNA1016085. The data generated and analyzed during the present study are available from the corresponding author upon reasonable request.
