# Supplementary material for: Impact of structurally diverse polysaccharides on colonic mucin O-glycosylation and gut microbiota
Source: NPJ Biofilms Microbiomes. 2023 Dec 11;9:97. doi: 10.1038/s41522-023-00468-3 (PMC10713555; doi:10.1038/s41522-023-00468-3)
Supplement: Supplementary file 1 — Supplementary-1 [file 41522_2023_468_MOESM1_ESM.pdf]

Supplementary Figures

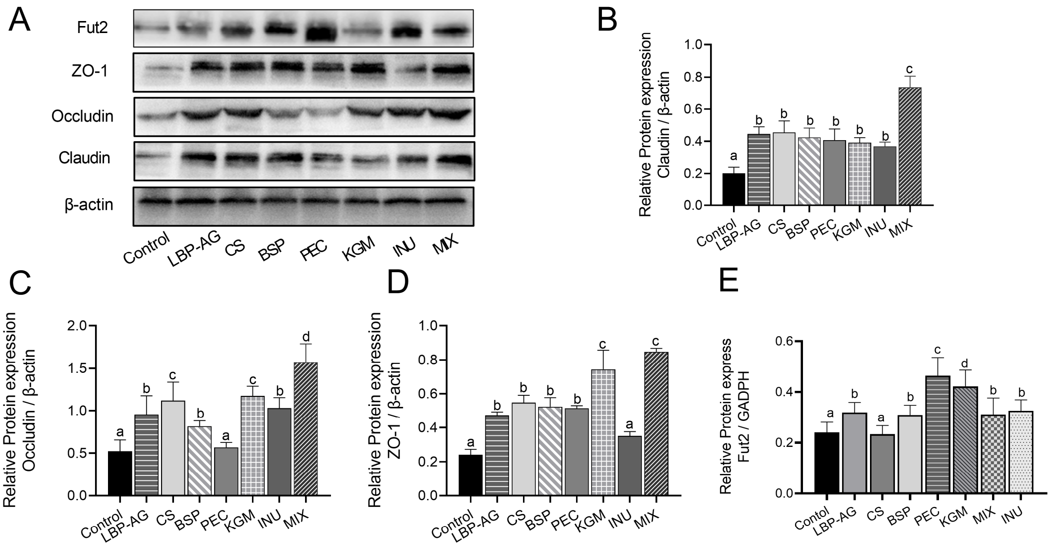

Supplementary Figure 1. Semi-quantitative determination of tight junction proteins and Fut2. (A) Representative western blots of tight junction proteins and Fut2, (B) claudin, (C) occludin, (D) ZO-1, and (E) Fut2 in the colonic tissue of mice from each group (mean ± SD, n = 5). Significance (*P < 0.05*) was determined by Duncan's test.

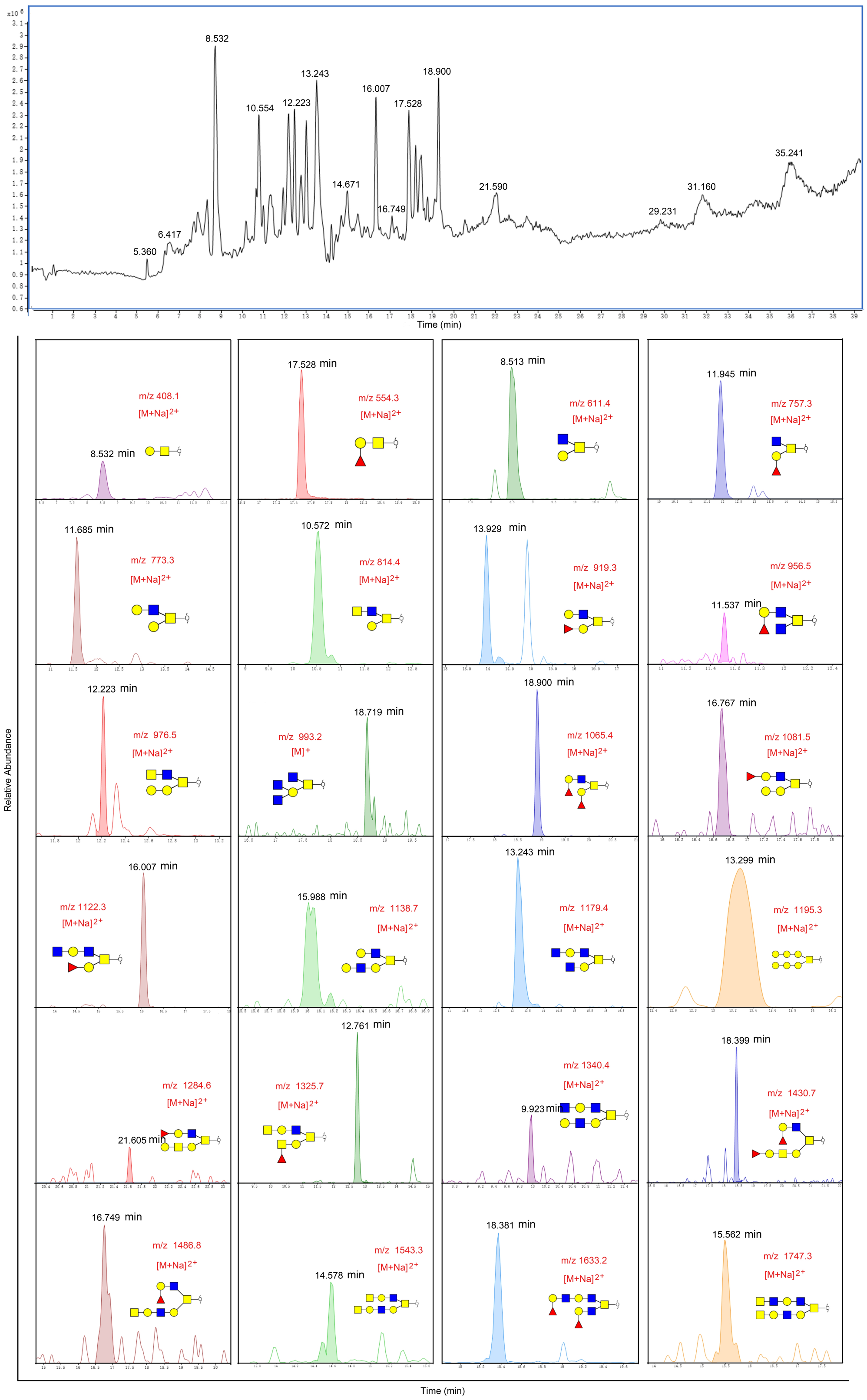

Supplementary Figure 2. Characterization of presumed neutral *O*-glycans. (A) Total Ion Chromatography and extracted ion chromatograms of neutral *O*-glycans from the CON group obtained in positive ion mode. (B) Spectrum of neutral *O*-glycans on colonic Muc2. Structural formulae: blue circle, glucose; yellow circle, galactose; blue square, N-acetylglucosamine; red triangle, fucose.

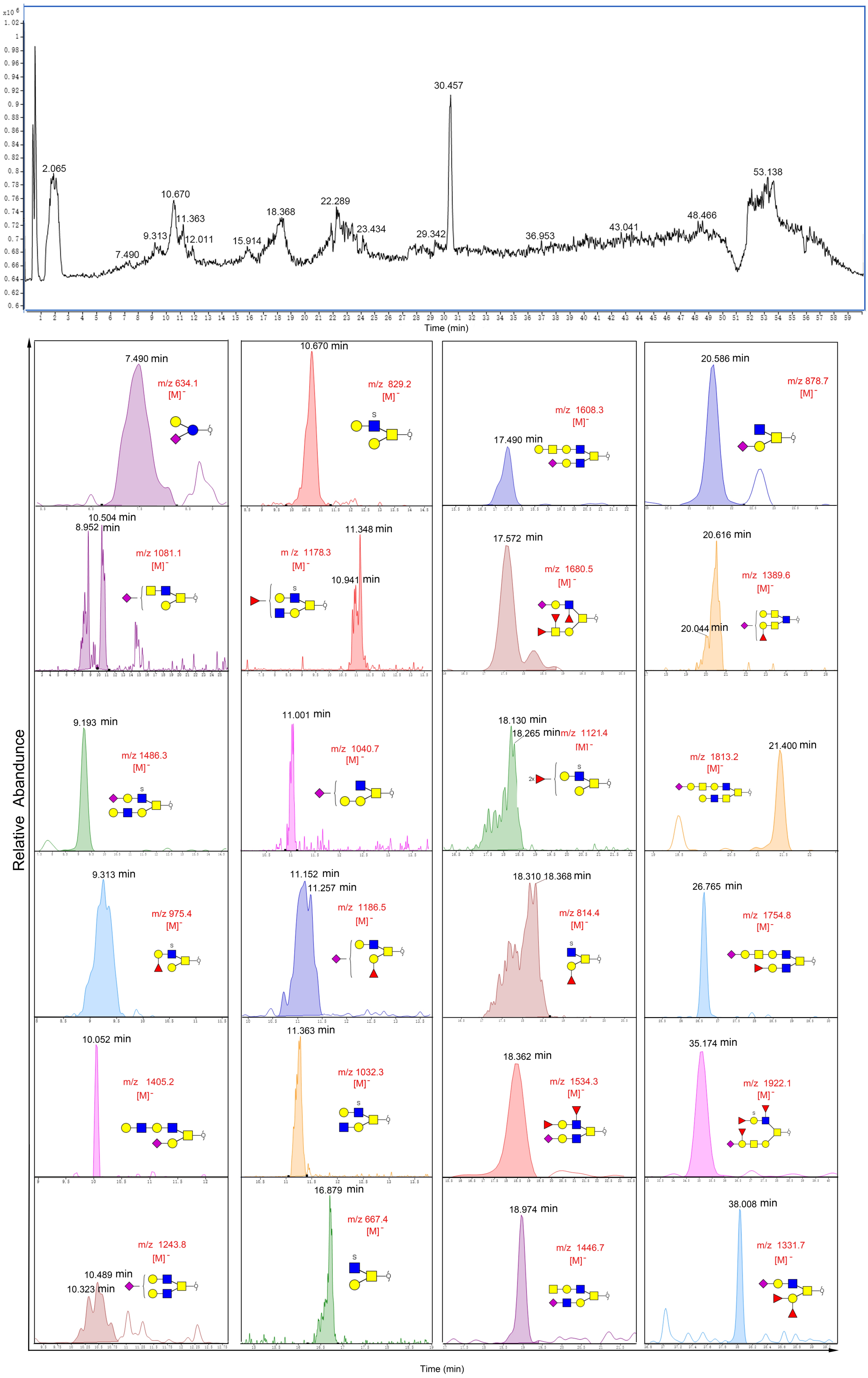

Supplementary Figure 3. Characterization of presumed sulphated and sialylated *O*-glycans. (A) Total Ion Chromatography and extracted ion chromatograms of sulphated and sialylated *O*-glycans from the CON group obtained in negative ion mode. (B) Spectrum of sulphated and sialylated *O*-glycans of colonic Muc2. Structural formulae: blue circle, glucose; yellow circle, galactose; blue square, N-acetylglucosamine; red triangle, fucose.

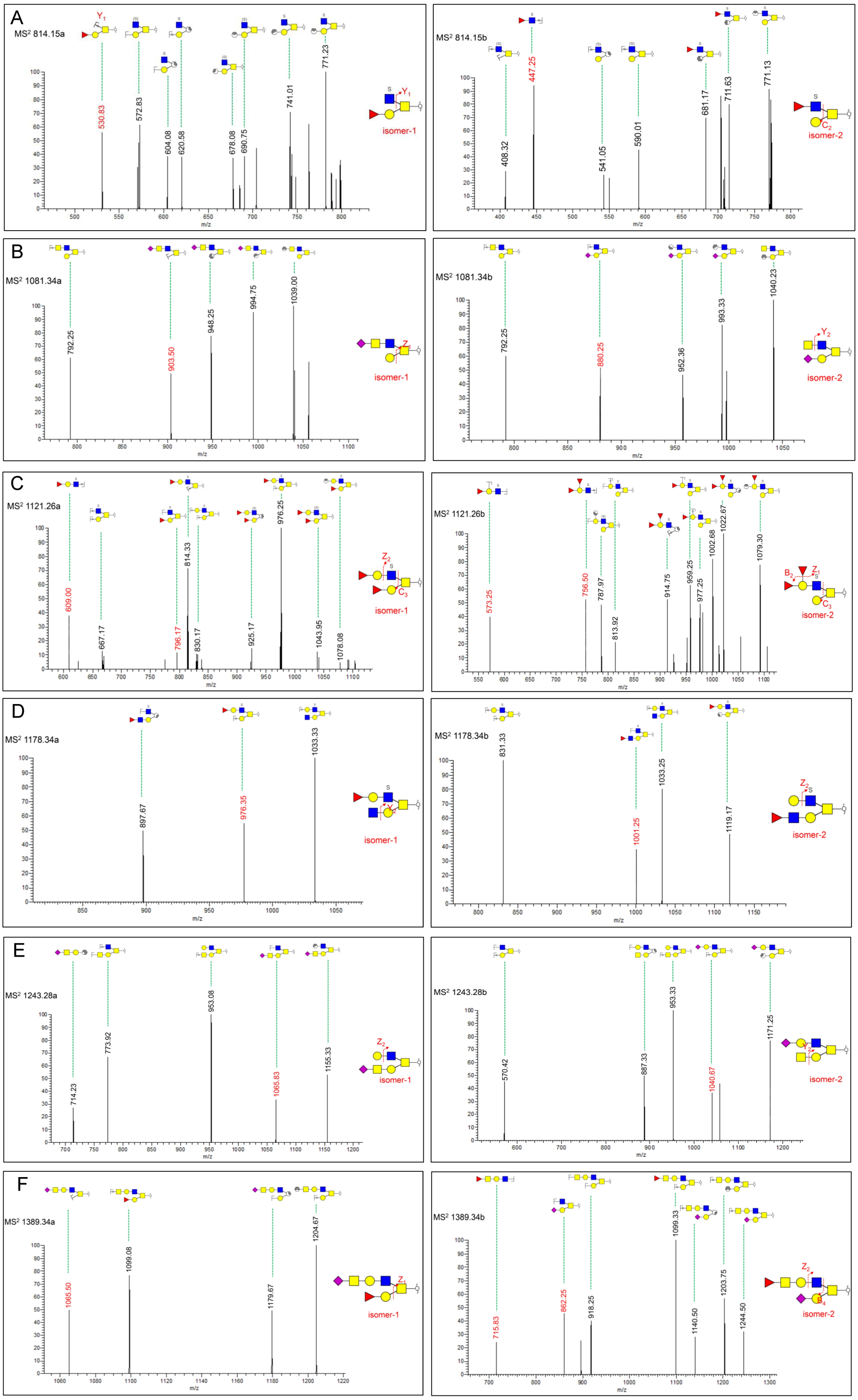

Supplementary Figure 4. Tandem MS spectra of isomers (A–F). Spectra for m/z 814 (A), 1081 (B), 1121 (C), 1178 (D), 1243 (E), and 1389 (F).

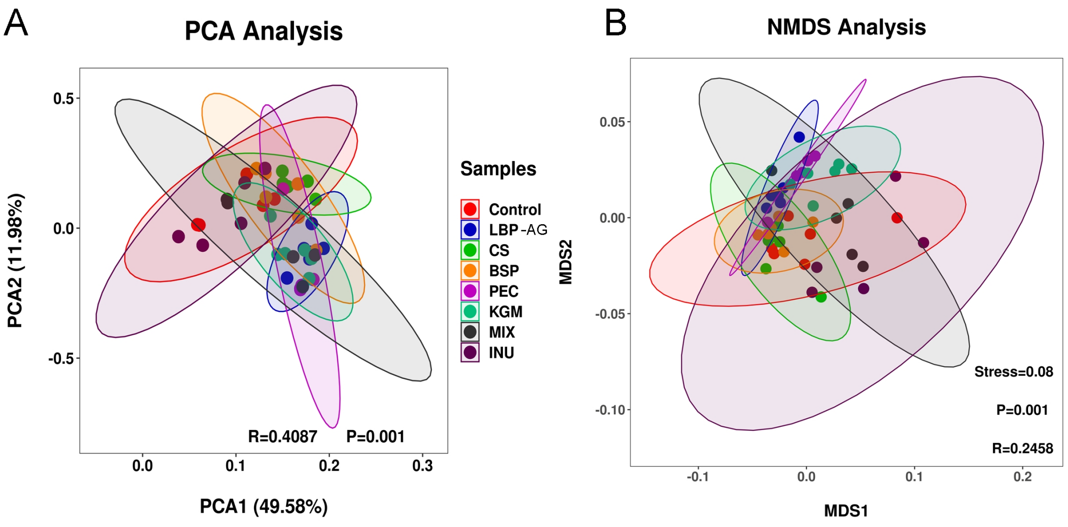

Con ***vs*** **LBP:** *P = 0.019*; **Con** ***vs*** **CS:** *P = 0.027*; **Con** ***vs*** **BSP:** *P = 0.046*; **Con** ***vs*** **PEC:** *P = 0.003*; **Con** ***vs*** **KGM:** *P = 0.007*; **Con** ***vs*** **MIX:** *P = 0.03*; **Con** ***vs*** **INU:** *P = 0.03*.

Supplementary Figure 5. The ordination of the gut microbiota. (A) Bray-Curtis similarity-based principal component analysis (*P=0.001*, PERMANOVA), (B) nMDS plot (*P=0.001*, PERMANOVA). Data analysed for PERMANOVA (n =6)

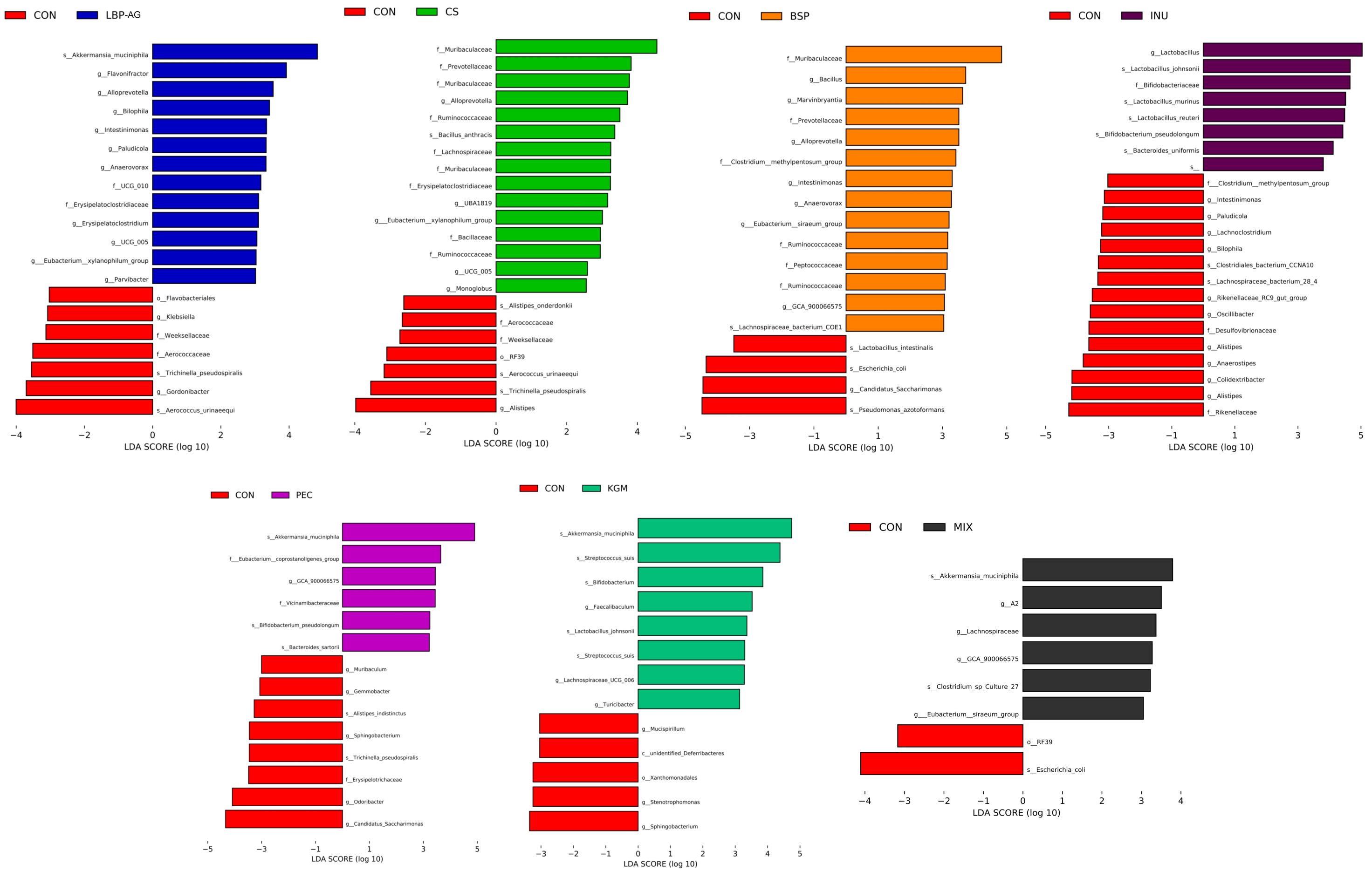

Supplementary Figure 6. LEfSe analyses. (A) CON *vs*. LBP-AG, (B) CON *vs*. CS, (C) CON *vs*. BSP, (D) CON *vs*. PEC, (E) CON *vs*. KGM, (F) CON *vs*. MIX, and (G) CON *vs*. INU groups. The histogram to the left shows LDA scores (LDA > 3) computed for each bacterial family, genus or species.

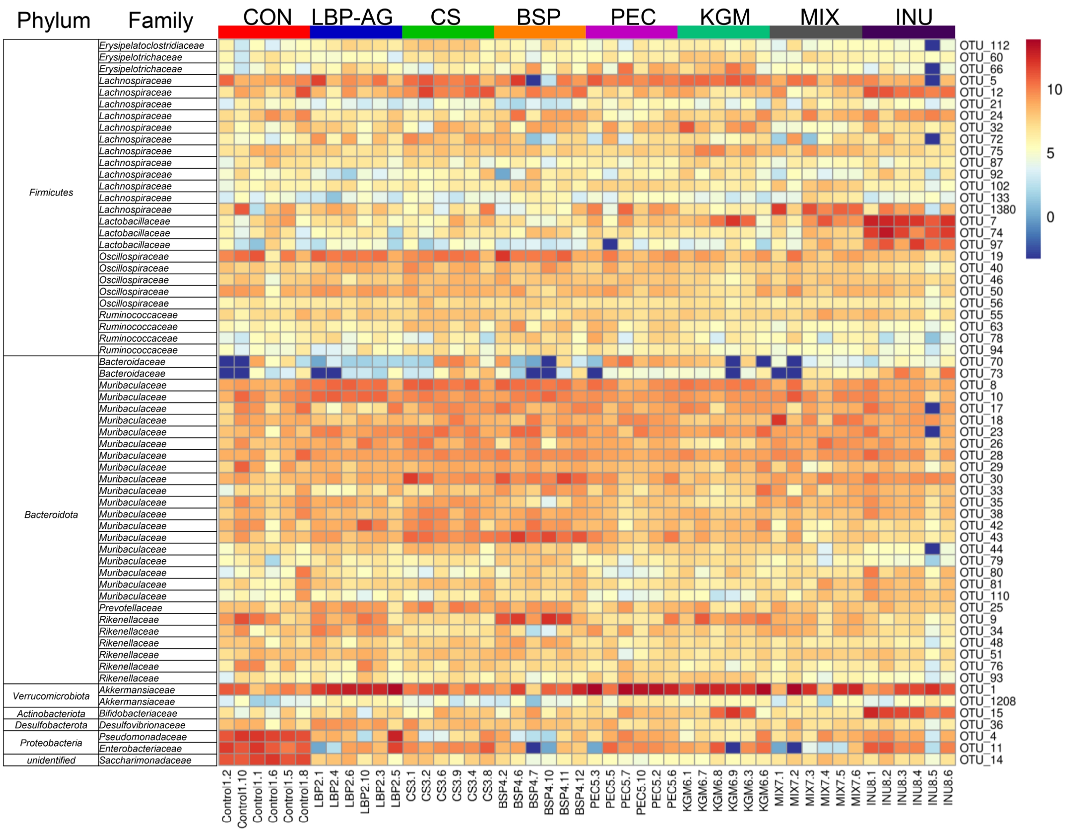

Supplementary Figure 7. Relative abundance (Log2 transformed) of OTUs in gut microbes displaying significant differences across polysaccharide groups (n = 6).

Supplementary Tables

Supplementary Table 1. Physicochemical properties of polysaccharide samples.

Samples	Monosaccharide composition	Molecular weight / kDa	sugar content / %	Sulphate content / %	protein content / %
LBP-AG	Ara:Gal=1:0.85	99.8	62.3±1.21	-	15.36±1.22
CS	GlcA:GalN=1:1.73	83.3	56.02±0.63	20.21±0.37	16.50±1.37
BSP	Fuc:Gal=1:0.5	34.8	81.12±1.02	18.01±1.27	12.26±0.87
PEC	Rha:Ara:Gal:GlcA=0.2:1:1:2	90.3	89.62±0.98	-	5.37±1.61
KGM	Man:Glc=1:0.5	6.2	91.13±1.46	-	2.8±0.81
INU	Fru:Glc=1:0.32	30.1	90.71±0.72	-	8.42±0.73

Supplementary Table 2. Nutritional composition of experimental diets.

Nutrient	Content(g/kg)
Moisture	100
Protein	201.8
Fat	46
Fiber	49
Ash	66
Ca	11.4
P	9.1

Feed ingredients included: corn, soybean meal, wheat bran, wheat flour, fish meal, salt, calcium bicarbonate, vitamin mixture, mineral mixture, and amino acids

Supplementary Table 3. Numbers of different *O*-glycans on mucin 2 in the mouse colon.

	Control	LBP-AG	CS	BSP	PEC	KGM	MIX	INU
Total *O*-glycans	54	52	53	54	53	53	53	51
The neutral *O*-glycans	24	22	23	24	24	24	24	24
The acid *O*-glycans	30	30	30	30	30	29	29	28
The non-fuc *O*-glycans	12	11	11	12	12	12	12	12
The fuc *O*-glycans	12	11	12	12	12	12	12	12
The sia *O*-glycans	18	18	18	18	18	17	17	17
The sul *O*-glycans	10	10	10	10	10	10	10	10
The sul and sia *O*-glycans	2	2	2	2	2	2	2	2
The Core 1 *O*-glycans	3	2	2	3	3	3	3	3
The Core 2 *O*-glycans	46	45	46	46	46	45	45	44
The Core 3 *O*-glycans	–	–	–	–	–	–	–	–
The Core 4 *O*-glycans	6	6	6	6	6	6	6	6

Supplementary Table 4. Spearman correlation coefficient (rho) of pair-wise comparisons between the relative abundance of gut bacteria and short-chain fatty acids ($P < 0.05$).

SCFAs	OTUs	rho	*p*-value	relation
Acetate	OTU19	0.7080	0.0493	positive
Acetate	OTU932	0.8198	0.0127	positive
Acetate	OTU40	0.7315	0.0391	positive
Acetate	OTU25	0.8308	0.0106	positive
Acetate	OTU10	0.8562	0.0066	positive
Acetate	OTU8	0.7480	0.0328	positive
Acetate	OTU1643	0.8895	0.0030	positive
Acetate	OTU96	0.9332	0.0007	positive
Acetate	OTU2	0.8717	0.0047	positive
Acetate	OTU1064	0.7492	0.0323	positive
Butyrate	OTU2363	0.7116	0.0477	positive
Butyrate	OTU1	0.8065	0.0155	positive
Butyrate	OTU1208	0.8252	0.0116	positive
Butyrate	OTU187	0.8084	0.0151	positive
Propionate	OTU1308	0.7258	0.0414	positive
Propionate	OTU1	0.7906	0.0194	positive
Propionate	OTU102	0.7743	0.0240	positive
Propionate	OTU1208	0.7341	0.0381	positive
Propionate	OTU187	0.7118	0.0476	positive

Supplementary Table 5. Putative structures and quantitative comparisons of *O*-glycans released from mucin 2 in the mouse colon.

Glycan Number	Mass (m/z)	Composition	Core	Proposed structure	Relative Abundance (peak area ×10^3) Mean±SEM							
					Control	LBP-AG	CS	BSP	PEC	KGM	MIX	INU
1	408	H1N1	1	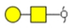	1.25±0.07a	2.48±0.47b	3.54±0.40c	1.33±0.14a	1.50±0.19a	1.21±0.16a	1.21±0.12a	1.51±0.28a
2	554	F1H1N1	1	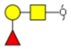	1.18±0.36a	15.5±0.51b	4.76±0.39c	1.64±0.30a	1.48±0.27a	1.43±0.22a	1.56±0.24a	1.33±0.22a
3	611	H1N2	2	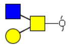	16.12±2.77a	36.93±2.12b	51.12±3.61c	35.22±3.58b	79.09±2.52d	77.65±3.32d	31.90±4.39b	61.10±4.59e
4	757	F1H1N2	2	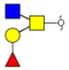	7.25±0.59a	3.86±0.14b	8.69±0.41c	6.88±0.74a	18.2±0.81d	9.68±0.99e	7.14±0.40a	6.19±0.22f
5	773	H2N2	2	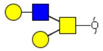	0.66±0.17a	1.34±0.22b	0.72±0.17a	1.53±0.18b	1.25±0.29b	1.37±0.05b	11.5±1.53c	1.70±0.20b
6	814	H1N3	2	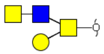	0.80±0.08a	0.73±0.11a	0.45±0.05b	1.04±0.23a	1.18±0.13c	0.69±0.11a	1.23±0.30c	1.39±0.26c
7	919	F1H2N2	2	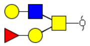	2.77±0.74a	0.97±0.14b	2.40±0.39a	1.81±0.22c	7.49±0.76d	5.77±0.43e	8.75±1.04d	5.75±0.93e
8	956	F1H1N3	4	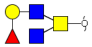	1.05±0.18a	1.20±0.18a	0.54±0.13b	1.22±0.29a	0.59±0.03b	0.73±0.07a	1.12±0.19a	0.73±0.14a
9	976	H2N3	2	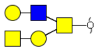	1.12±0.11a	3.91±0.09b	1.50±0.19c	1.93±0.13c	16.5±2.22d	1.10±0.16a	1.48±0.31a	1.60±0.17c
10	993	H1N4	2	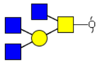	0.97±0.16a	1.00±0.11a	0.56±0.18b	1.71±0.68c	1.98±0.11c	1.13±0.15a	1.65±0.08c	0.69±0.12a
11	1065	F2H2N2	2	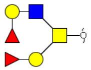	2.96±0.64a	5.34±0.40b	2.60±0.22a	4.46±0.65b	14.6±0.80c	10.3±1.30d	12.6±1.97d	10.8±1.15d
12	1081	F1H3N2	2	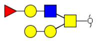	2.09±0.25a	0.90±0.20b	0.57±0.11c	2.09±0.31a	1.66±0.32a	1.61±0.25a	1.33±0.13b	1.09±0.19b

Glycan Number	Mass (m/z)	Composition	Core	Proposed structure	Relative Abundance (peak area ×10^3) Mean±SEM							
					Control	LBP-AG	CS	BSP	PEC	KGM	MIX	INU
13	1122	F1H2N3	2	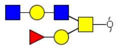	1.65±0.28a	1.20±0.17a	0.59±0.10b	1.39±0.30a	1.24±0.10a	1.34±0.31a	1.59±0.21a	1.30±0.20a
14	1138	H3N3	2	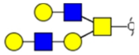	2.34±0.30a	0.86±0.09b	1.42±0.10c	1.99±0.22a	3.33±1.02d	1.49±0.20c	1.43±0.29c	1.37±0.32c
15	1179	H2N4	2	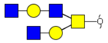	2.54±0.30a	2.03±0.26a	1.55±0.31b	2.53±0.17a	2.04±0.27a	1.72±0.19b	2.15±0.26a	2.16±0.31a
16	1195	H6N1	1	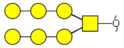	1.15±0.22a	-	-	1.58±0.19b	1.68±0.34b	1.62±0.19b	2.55±0.15c	1.00±0.19a
17	1284	F1H3N3	2	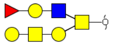	0.81±0.13a	-	0.45±0.10b	1.70±0.27c	0.51±0.03b	0.92±0.09a	1.46±0.32c	1.61±0.34c
18	1325	F1H2N4	2	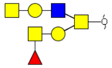	1.05±0.22a	0.54±0.08b	0.59±0.07b	1.53±0.39a	1.07±0.21a	1.70±0.47a	1.38±0.28a	1.49±0.13a
19	1340	H3N4	2	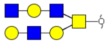	0.87±0.10a	0.62±0.11a	0.62±0.18a	1.57±0.15b	1.67±0.17b	2.09±0.41b	1.25±0.25b	1.35±0.27b
20	1430	F2H3N3	2	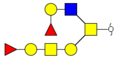	1.89±0.22a	0.46±0.01b	0.58±0.04b	1.77±0.47a	1.03±0.14c	1.64±0.27a	1.31±0.11a	1.35±0.19a
21	1486	F1H3N4	2	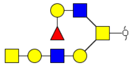	0.88±0.16a	0.36±0.00b	0.47±0.11b	3.60±0.56c	1.90±0.61d	0.99±0.08a	1.95±0.51d	1.71±0.16d
22	1543	H3N5	2	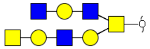	1.17±0.24a	0.59±0.05b	0.95±0.14a	2.72±0.57c	0.95±0.20a	1.87±0.27d	2.07±0.27d	1.28±0.22a
23	1633	F2H3N4	4	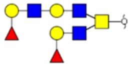	1.23±0.26a	0.83±0.01b	1.58±0.20a	2.18±0.58c	1.45±0.26a	1.89±0.20c	2.28±0.23c	1.72±0.29a
24	1747	H3N6	2	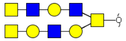	1.12±0.25a	0.77±0.13a	0.50±0.05b	1.56±0.17c	0.91±0.04a	1.33±0.32a	2.30±0.14d	1.46±0.19a

Glycan Number	Mass (m/z)	Composition	Core	Proposed structure	Relative Abundance (peak area ×10^3) Mean±SEM							
					Control	LBP-AG	CS	BSP	PEC	KGM	MIX	INU
25	667	S1H1N2	2	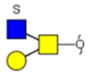	10.64±0.24a	4.75±0.19b	5.09±0.51b	6.35±0.67b	5.90±0.50b	5.21±0.61b	1.37±0.02c	3.94±0.11d
26	814a	S1F1H1N2	2	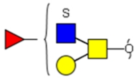	10.66±0.28a	6.01±0.39b	6.39±0.77b	6.32±0.55b	6.54±0.63b	5.48±0.73b	1.69±0.16c	7.50±0.25b
27	814b				5.09±1.00a	1.91±0.37b	2.58±0.50b	2.67±0.52b	4.31±0.84a	5.44±1.06a	4.37±0.85a	2.72±0.53b
28	829	S1H2N2	2	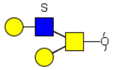	2.07±0.07a	1.15±0.04b	1.53±0.13c	1.17±0.08b	0.41±0.01d	0.55±0.05d	0.58±0.02d	0.42±0.13d
29	878	H1N2A1	2	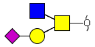	3.10±0.26a	0.73±0.07b	0.40±0.10c	0.84±0.15b	1.56±0.17d	0.12±0.01e	0.34±0.01c	1.39±0.11d
30	975	S1F1H2N2	2	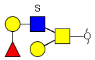	6.80±0.14a	10.28±1.08b	12.32±0.76b	5.60±0.83a	4.93±0.77a	5.53±1.17a	6.60±0.33a	4.84±0.42a
31	1032	S1H2N3	4	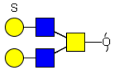	1.49±0.06a	2.12±0.20b	1.70±0.35a	1.02±0.15c	0.53±0.09d	0.36±0.07d	1.62±0.19a	1.34±0.09a
32	1040	H2N2A1	2	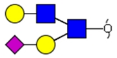	1.95±0.27a	2.70±0.18b	1.34±0.10c	1.37±0.08c	1.08±0.15c	0.51±0.07d	0.64±0.05d	1.40±0.04c
33	1081a	H1N3A1	2	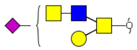	1.66±0.07a	1.74±0.06a	1.00±0.13b	1.36±0.07b	0.75±0.06b	0.25±0.03c	0.72±0.04b	0.84±0.02d
34	1081b				1.36±0.07a	1.44±0.06a	5.93±0.40b	2.08±0.11c	6.14±0.09b	0.29±0.03d	7.27±0.02e	0.52±0.07f
35	1121a	S1F2H2N2	2	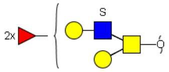	2.52±0.03a	5.47±0.60b	2.52±0.15a	2.91±0.11a	6.00±0.38b	2.99±0.25a	5.33±0.17b	4.93±0.23b
36	1121b				2.20±0.06a	7.32±0.97b	10.2±0.46c	5.97±0.38b	2.74±0.25d	3.66±0.18e	1.65±0.06f	1.66±0.07f
37	1178a	S1F1H2N3	2	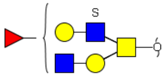	1.91±0.02a	1.70±0.35a	1.23±0.06b	1.22±0.24b	1.08±0.15b	0.83±0.11c	1.26±0.05b	0.63±0.06d
38	1178b				1.60±0.32a	1.27±0.08a	1.25±0.47a	1.75±0.41a	1.14±0.24a	1.14±0.19a	0.57±0.13b	2.22±0.43a
39	1186a	F1H2N2A1	2	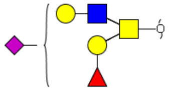	0.76±0.03a	0.85±0.10a	1.03±0.19a	0.78±0.09a	0.50±0.07b	0.26±0.04c	0.66±0.07a	0.47±0.02b
40	1186b				1.36±0.11a	1.23±0.13a	0.89±0.19b	1.09±0.03b	0.97±0.09b	0.60±0.17c	1.52±0.12a	0.16±0.01d

Glycan Number	Mass (m/z)	Composition	Core	Proposed structure	Relative Abundance (peak area ×10^3) Mean±SEM							
					Control	LBP-AG	CS	BSP	PEC	KGM	MIX	INU
41	1243a	H2N3A1	4	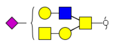	2.14±0.18a	0.84±0.11b	1.28±0.31c	0.96±0.13c	0.91±0.07c	1.21±0.11c	0.39±0.05d	0.93±0.02c
42	1243b				1.63±0.02a	2.21±0.10b	1.13±0.22c	0.91±0.07c	0.46±0.03d	0.39±0.08d	0.77±0.06c	0.80±0.06c
43	1331	F2H2N2A1	2	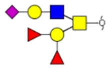	0.79±0.05a	0.57±0.02b	0.52±0.11b	0.64±0.11b	0.41±0.05b	0.16±0.03c	0.53±0.08b	-
44	1389a	F1H3N2A1	2	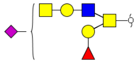	1.50±0.19a	2.87±0.23b	1.15±0.25a	1.46±0.09a	1.16±0.18a	0.95±0.14c	2.23±0.15d	0.57±0.02e
45	1389b				1.54±0.16a	0.91±0.07b	0.79±0.08b	0.46±0.03c	0.35±0.05c	0.14±0.01d	0.41±0.04c	0.34±0.02c
46	1405	H3N3A1	2	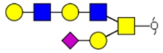	1.63±0.15a	2.83±0.14b	2.34±0.59b	3.05±0.16b	2.28±0.46b	2.77±0.86b	1.32±0.13c	3.68±0.35b
47	1446	H2N4A1	2	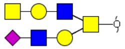	1.34±0.13a	0.79±0.08b	0.45±0.09c	1.13±0.09a	0.71±0.06b	-	-	-
48	1486	S1H3N3A1	2	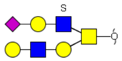	1.36±0.15a	0.80±0.10b	0.45±0.10c	1.14±0.17a	1.12±0.10a	0.25±0.02d	0.58±0.00e	-
49	1534	F2H2N3A1	4	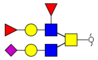	0.80±0.18a	0.78±0.07a	0.40±0.12b	0.22±0.09c	0.60±0.07a	0.18±0.01c	0.53±0.04b	0.51±0.03b
50	1608	H3N4A1	4	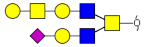	1.60±0.16a	0.67±0.06b	0.43±0.21b	1.01±0.20c	1.44±0.09a	0.25±0.06d	0.46±0.11b	0.29±0.02d
51	1680	F3H2N3A1	2	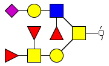	1.29±0.17a	1.06±0.08a	0.49±0.14b	0.88±0.04c	0.55±0.08b	0.30±0.01d	0.65±0.04b	0.18±0.02e
52	1754	F1H3N4A1	4	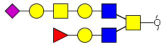	1.22±0.37a	0.75±0.12a	0.36±0.11b	1.02±0.22a	1.19±0.21a	0.17±0.03b	0.37±0.02b	0.31±0.03b
53	1813	H3N5A1	2	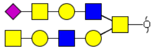	0.77±0.14a	0.56±0.05a	0.30±0.14b	0.86±0.19a	0.58±0.19a	0.14±0.02b	0.47±0.10a	0.37±0.06b
54	1922	S1F3H3N3A1	2	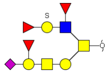	0.87±0.16a	0.75±0.13a	0.23±0.01b	0.67±0.22a	0.42±0.12c	0.11±0.03d	0.32±0.07c	0.33±0.07c

Note: Significance (*P < 0.05*) was determined using Turkey's test (n = 5).
